# Significance of information obtained during transanal drainage tube placement after anterior resection of colorectal cancer

**DOI:** 10.1371/journal.pone.0271496

**Published:** 2022-08-29

**Authors:** Yuki Okazaki, Masatsune Shibutani, Hisashi Nagahara, Tatsunari Fukuoka, Yasuhito Iseki, En Wang, Kiyoshi Maeda, Kosei Hirakawa, Masaichi Ohira

**Affiliations:** 1 Department of Gastroenterological Surgery, Osaka City University Graduate School of Medicine, Osaka, Japan; 2 Department of Gastroenterological Surgery, Osaka City General Hospital, Osaka, Japan; Shuguang Hospital, CHINA

## Abstract

**Introduction:**

It has recently been reported that the placement of a transanal drainage tube after rectal cancer surgery reduces the rate of anastomotic leakage. However, transanal drainage tube cannot completely prevent anastomotic leakage and the management of transanal drainage tube needs to devise. We investigated the information obtained during transanal drainage tube placement and evaluated the relationship between these factors and anastomotic leakage.

**Patients and methods:**

Fifty-one patients who underwent anterior resection of rectal cancer was retrospectively reviewed. transanal drainage tube was placed for more than 5 days after surgery. The daily fecal volume from transanal drainage tube was measured on postoperative day 1–5, and the defecation during transanal drainage tube placement was investigated.

**Results:**

Anastomotic leakage during transanal drainage tube placement occurred in 4 patients. The anastomotic leakage rate during transanal drainage tube placement in patients whose maximum daily fecal volume or total fecal volume from the transanal drainage tube during postoperative days 1–5 was large was significantly higher than that in patients whose fecal volume was small. The anastomotic leakage rate of the patients with intentional defecation during transanal drainage tube placement was significantly higher than that of the patients without intentional defecation during transanal drainage tube placement. The maximum daily fecal volume and the total fecal volume from the transanal drainage tube during postoperative days 1–5 in patients who experienced intentional defecation during transanal drainage tube placement was significantly higher than that of patients without intentional defecation during transanal drainage tube placement.

**Conclusion:**

A large fecal volume from transanal drainage tube after anterior rectal resection or intentional defecation in patients with transanal drainage tube placement were suggested to be risk factors for anastomotic leakage.

## Introduction

Anastomotic leakage after the resection of colorectal cancer is a serious complication that is associated with short-term outcomes, such as reoperation, extension of hospital stay, and increased perioperative mortality [1–5], as well as long-term oncological effects, such as a poor prognosis due to local recurrence [6–8]. The anastomotic leakage rate after resection of rectal cancer is higher in comparison to other colon cancers [9]. Thus, in order to prevent anastomotic leakage after low-anterior resection (LAR) of the rectum, various methods have been adapted, such as adequate mobilization of the colon [10], the use of intracorporeal reinforcing sutures [11] and evaluation of the blood flow by fluorescence imaging with indocyanine green (ICG) [12]. In addition, in cases in which there is considered to be a high risk of anastomotic leakage, such as cases with anastomosis at a low rectal position, diverting stomas can be constructed to reduce the burden of anastomosis [13]. Recently, it has been reported that the placement of a transanal drainage tube (TDT), which is technically easy and which can economically decompress the anastomotic site [14], is effective for preventing anastomotic leakage after rectal cancer surgery [15–17]. However, even if a TDT is used, defecation may occur that does not pass through the TDT, and anastomotic leakage may occur. Furthermore, the timing of the removal of the TDT was sometimes delayed based on the judgment of each surgeon. Thus, there may be room for improving the method of managing TDT in the perioperative period. The present study therefore explored the mechanism underlying the occurrence of anastomotic leakage despite using a TDT by evaluating the association between the perioperative clinical information obtained during TDT placement and anastomotic leakage and suggested a strategy for preventing anastomotic leakage. Thus, we retrospectively evaluated the association between clinical information during TDT placement and anastomotic leakage.

## Patients and methods

Fifty-one consecutive patients underwent surgery for the treatment of colorectal cancer with the double staple technique (DST) and who underwent TDT placement for 5 days or more after surgery, at Osaka City University Hospital between January 2016 and March 2019. None of the 51 patients underwent construction of a diverting stoma. Patients treated with preoperative chemotherapy or chemoradiotherapy, and those who underwent decompression treatment for intestinal obstruction were excluded from the present study. The characteristics and clinical information of the 51 patients were retrospectively based on their electronic medical records. The associations between anastomotic leakage and preoperative risk factors for anastomotic leakage, such as male sex, advanced age, obesity, high primary T/N stage, large-diameter tumor and anastomosis at a low position [18–21] were evaluated. The World Health Organization has reported that the body mass index (BMI; weight / length^2^) ≥25.0 kg/m^2^ indicates an overweight status [22]. We therefore set 25.0 kg/m^2^ as the cut-off value of the BMI. The primary pathological T/N stage was defined by the Union for International Cancer Control (UICC) 8th edition [23]. The patients were classified into T1-3 and T4 groups or N0 and N1-3 groups. They were also divided into high-anterior resection (HAR) and LAR groups. The tumor diameter was determined based on the maximum length of the tumor. Tumor diameter was defined as 0 after endoscopic treatment. The cut-off value for the tumor diameter was calculated based on the receiver operating characteristic (ROC) curve.

The standard mechanical bowel preparation at our hospital was fasting after lunch and the internal use of polyethylene glycol solution at 14:00 on the day before surgery. However, two patients had diarrhea before the mechanical bowel preparation. No patients received antibiotic prophylaxis. For all the patients, a 10-mm Pleats drain (Akita Sumitomo Bakelite, Japan) was inserted from the anus and positioned with the tip approximately 5 cm above the anastomotic site at the last step of the colorectal cancer operation under general anesthesia. The removal of the TDT was scheduled for postoperative day (POD) 5; however, removal was sometimes delayed depending on the judgment of each surgeon.

The daily fecal volume from TDT and the total fecal volume for the 5 days of TDT placement (PODs 1–5 after the resection for CRC) were measured.

The cut-off values of the fecal volume were calculated based on an ROC curve analysis in order to determine the relationship between anastomotic leakage and the fecal volume from the TDT. The patients were divided to two groups: the high-volume group and the low-volume group.

The fecal matter that did not pass through the TDT during POD 1–5 was investigated from electronic medical records. In this study, the defecation that patients consciously performed during TDT placement was defined as intentional defecation, while the discharge that flowed outside the TDT unconsciously was defined as fecal incontinence. We evaluated the associations between anastomotic leakage and intentional defecation and between anastomotic leakage and fecal incontinence.

In addition, a subgroup analysis of patients in whom no anastomotic leakage occurred during TDT placement was performed. The association between the fecal volume from the TDT at POD 5, when TDT removal was scheduled, and the anastomotic leakage after removal of TDT was evaluated.

The details of anastomotic leakage were collected from the medical records of each surgeon. Anastomotic leakage was defined by major leakage (e.g., fecal discharge from the abdominal drain tube or the discharging of contrast agent into the abdominal cavity during fluoroscopic examination) and by minor leakage (e.g., free air around the anastomotic site on CT after the patient presented fever or abdominal pain).

All of the statistical analyses were performed using JMP 14.2.0 (SAS Institute, Japan, Tokyo). The chi-squared test, the Fisher’s exact test and Mann-Whitney U-test were used to analyze the significance of associations between 2 groups. P values of <0.05 were considered to indicate statistical significance. Baseline variables with p values of <0.10 on the univariate analysis were included as covariates in the multivariate logistic regression analysis. P values of <0.05 were considered to indicate statistical significance.

This retrospective study was approved by the Ethics Committee of Osaka City University (approval number: 4182) and conducted in accordance with the Declaration of Helsinki. All patients provided their written informed consent.

## Results

### Patient characteristics

The patient characteristics are listed in Table 1. Of the 51 patients who were analyzed, 32 were male and 19 were female. The median age was 70 years (range: 41–87). The median BMI was 23.8 kg/m^2^ (range: 15.4–33.5). The pathological T stage of 47 patients was T1-3, and that of 4 patients was T4. The pathological N stage of 43 patients was N0, and that of 8 patients was N1-3. The median tumor diameter was 30.0 mm (range: 0–100.0 mm). LAR was performed for 23 patients and HAR was performed for 28 patients. Laparoscopic operations were performed for 48 patients and open surgery was performed for 3 patients.

**Table 1:** Patient characteristics.

Clinical factor	n = 51
Gender, n(%)	
Male	32 (62.7%)
Female	19 (37.3%)
Age (years)	
Median (range)	70 (41–87)
BMI (kg/m^2^)	
Median (range)	23.8 (15.4–33.5)
Pathologic T stage	
T1	18 (35.3%)
T2	6 (11.8%)
T3	23 (45.1%)
T4	4 (7.8%)
Pathologic N stage	
N0	43 (84.3%)
N1	5 (9.8%)
N2	2 (3.9%)
N3	1 (2.0%)
Diameter of tumor (mm)	
Median (range)	30.0 (0–100.0)
Operative method, n(%)	
High-anterior resection	28 (54.9%)
Low-anterior resection	23 (45.1%)
Surgical approach, n(%)	
Laparoscopic surgery	48 (94.1%)
Open surgery	3 (5.9%)

BMI: Body Mass Index

### Preoperative factors associated with anastomotic leakage

The median tumor diameter of the patients without anastomotic leakage was 28.0mm (range 0 to 100.0; interquartile range 15.0 to 43.0). The median tumor diameter of the patients who experienced anastomotic leakage was 55.0mm (range 35.0 to 80.0; interquartile range 40.0 to 73.8). The tumor diameter of the patients who experienced anastomotic leakage was significantly greater than that of the patients without anastomotic leakage (p = 0.021) (S1 Fig).

We used the tumor diameter, which was a continuous variable, as the test variable and the occurrence of anastomotic leakage as the state variable. When we investigated the cut-off value for the tumor diameter using the ROC curve, we found that the appropriate cut-off value for the tumor diameter was 35.0 mm (sensitivity of 87.5%; specificity of 67.4%) (S2 Fig). We therefore set 35.0 mm as the cut-off value and classified patients into high and low groups based on this value.

The anastomotic leakage rate was significantly higher in the groups with LAR and ≥35.0 mm tumor diameter than in the other groups (p = 0.016, p = 0.006, respectively) (Table 2).

**Table 2:** Preoperative factors associated with anastomotic leakage.

	Anastomotic leakage		
Preoperative factor	Negative (n = 43)	Positive (n = 8)	p-value
Gender, n(%)			
Male	27 (62.8%)	5 (62.5%)	>0.999
Female	16 (37.2%)	3 (37.5%)	
Age(years)	70	62.5	0.161
Median(range)	(41–87)	(51–80)	
BMI, n(%)			
≥25.0kg/m^2^	17 (39.5%)	2 (25.0%)	0.694
<25.0kg/m^2^	26 (60.5%)	6 (75.0%)	
Pathological T stage			
≤T3	40 (93.0%)	7 (87.5%)	0.506
T4	3 (7.0%)	1 (12.5%)	
Pathological N stage			
N0	36 (83.5%)	7 (87.5%)	>0.999
≥N1	7 (16.5%)	1 (12.5%)	
Diameter of tumor, n(%)			
≥35.0 mm	14 (32.6%)	7 (87.5%)	0.006
<35.0 mm	29 (67.4%)	1 (12.5%)	
Operative method, n(%)			
High-anterior resection	27 (62.8%)	1 (12.5%)	0.016
Low-anterior resection	16 (37.2%)	7 (87.5%)	

BMI: Body Mass Index

### Occurrence and timing of anastomotic leakage

Anastomotic leakage occurred in 8 patients (15.7%). Four of these 8 cases occurred during TDT placement, and 4 of the 8 cases occurred after the removal of the TDT. Major leakage occurred in 2 patients (3.9%), and minor leakage occurred in 6 patients (11.8%). Re-operation for anastomotic leakage was performed in the 2 major leakage patients (3.9%). Of the four cases of anastomotic leakage during TDT placement, major leakage occurred in two patients, and minor leakage occurred in the other two. All four instances of anastomotic leakages after the removal of the TDT were minor.

### Association between the fecal volume from the TDT during POD 1–5 and the anastomotic leakage during TDT placement

**Fig 1:**
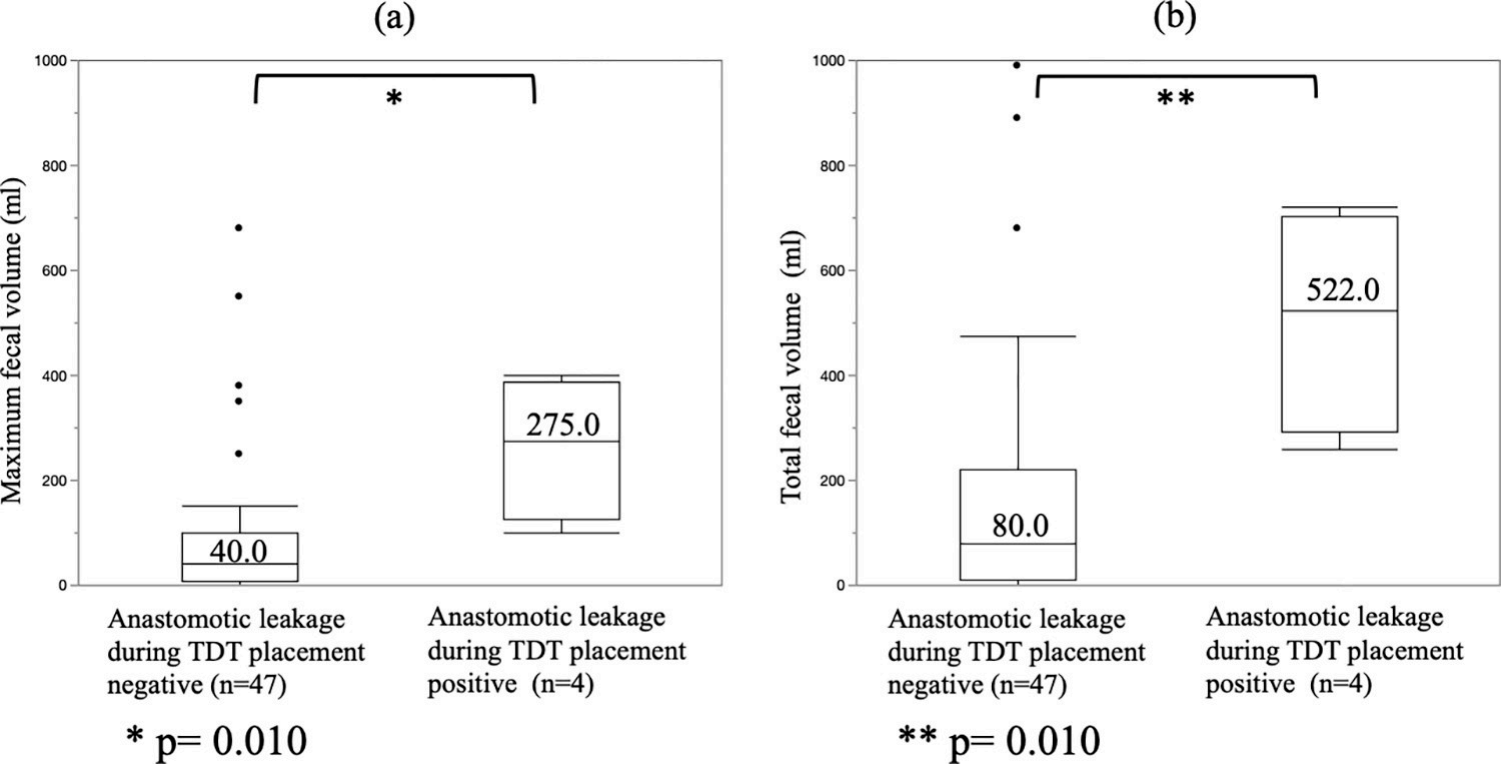
Association between anastomotic leakage during transanal drainage tube placement and the fecal volume from the transanal drainage tube. (a) The anastomotic leakage during transanal drainage tube (TDT) placement-positive group had a significantly greater maximum daily fecal volume during POD 1–5 than the anastomotic leakage during TDT placement-negative group (median total fecal volume: 275.0 ml vs. 40.0 ml, respectively. p = 0.010). (b) The anastomotic leakage during TDT placement-positive group had a significantly greater total fecal volume during POD 1–5 than the anastomotic leakage during TDT placement-negative group (median total fecal volume: 522.0 ml vs. 80.0 ml, respectively. p = 0.010).

The median maximum fecal volume from the TDT during POD 1–5 in patients who experienced anastomotic leakage during TDT placement was 275.0ml (range 100.0 to 400.0; interquartile range 125.0 to 388.0). The median maximum fecal volume from the TDT during POD 1–5 of the patients without anastomotic leakage during TDT placement was 40.0ml (range 0 to 680.0; interquartile range 8.0 to 100.0). The maximum fecal volume from the TDT during POD 1–5 in patients who experienced anastomotic leakage during TDT placement was significantly greater than that of the patients without anastomotic leakage during TDT placement (p = 0.010) (Fig 1A). The median total fecal volume from the TDT during POD 1–5 in patients who experienced anastomotic leakage during TDT placement was 522.0ml (range 260.0 to 720.0; interquartile range 292.5 to 703.8). The median total fecal volume from the TDT during POD 1–5 of the patients without anastomotic leakage during TDT placement was 80.0ml (range 0 to 1970.0; interquartile range 9.0 to 220.0). The total fecal volume from the TDT during POD 1–5 in patients who experienced anastomotic leakage during TDT placement was significantly higher than that of the patients without anastomotic leakage during TDT placement (p = 0.010) (Fig 1B).

We used the maximum daily fecal volume from the TDT during PODs 1–5, which was a continuous variable, as the test variable and the occurrence of anastomotic leakage during the TDT placement as the state variable. When we investigated the cut-off value for the maximum daily fecal volume from the TDT during PODs 1–5 using the ROC curve, we found that the appropriate cut-off value for the maximum daily fecal volume was 100.0 ml (sensitivity of 100.0%; specificity of 74.5%) (S3A Fig). Using the ROC curve in the same manner, we set the cut-off value for the total fecal volume from the TDT during PODs 1–5 at 260.0 ml (sensitivity of 100.0%; specificity of 83.0%) (S3B Fig). We therefore set each of these values of fecal volume as the relevant cut-off values and classified patients into the high and low groups.

The anastomotic leakage rate during TDT placement in patients in whom the maximum daily fecal volume from the TDT during POD 1–5 was ≥100.0 ml was significantly higher than that of the patients in whom the maximum daily fecal volume from the TDT during POD 1–5 was <100.0 ml (p = 0.007). The anastomotic leakage rate during TDT placement in patients in whom the total fecal volume from the TDT during POD 1–5 was ≥260.0 ml was significantly higher than that of the patients in whom the total fecal volume from the TDT during POD 1–5 was <260.0 ml (p = 0.002) (Table 3).

**Table 3:** Association between the fecal volume from the transanal drainage tube during postoperative day 1 to 5 and the anastomotic leakage during placement of transanal drainage tube.

	Anastomotic leakage during TDT placement		p-value
	Negative (n = 47)	Positive (n = 4)	
Maximum daily fecal volume from TDT during POD1 to 5, n(%)			
≥100.0 ml	12 (25.5%)	4 (100.0%)	0.007
<100.0 ml	35 (74.5%)	0 (0%)	
Total fecal volume from TDT during POD1 to 5, n(%)			
≥260.0 ml	8 (11.6%)	4 (100.0%)	0.002
<260.0 ml	39 (88.4%)	0 (0%)	

TDT: Transanal drainage tube, POD: postoperative day

### Association between fecal discharge not through TDT and the anastomotic leakage during TDT placement

The anastomotic leakage rate of the patients who experienced fecal incontinence during TDT placement was not significantly different from that in patients without fecal incontinence during TDT placement. However, the anastomotic leakage rate of patients who experienced intentional defecation during TDT placement was significantly higher than that of patients without intentional defecation during TDT placement (p = 0.028) (Table 4).

**Table 4:** Association between the anastomotic leakage during placement of transanal drainage tube and fecal discharge not through transanal drainage tube during transanal drainage tube placement.

	Anastomotic leakage during TDT placement		p-value
	Negative (n = 47)	Positive (n = 4)	
Fecal incontinence, n(%)			
	42 (89.4%)	4 (100.0%)	>0.999
Yes	5 (10.6%)	0 (0%)	
Intentional defecation, n(%)			
No	39 (83.0%)	1 (25.0%)	0.028
Yes	8 (17.0%)	3 (75.0%)	

TDT: transanal drainage tube

### Association between intentional defecation during TDT placement and the fecal volume from TDT

**Fig 2:**
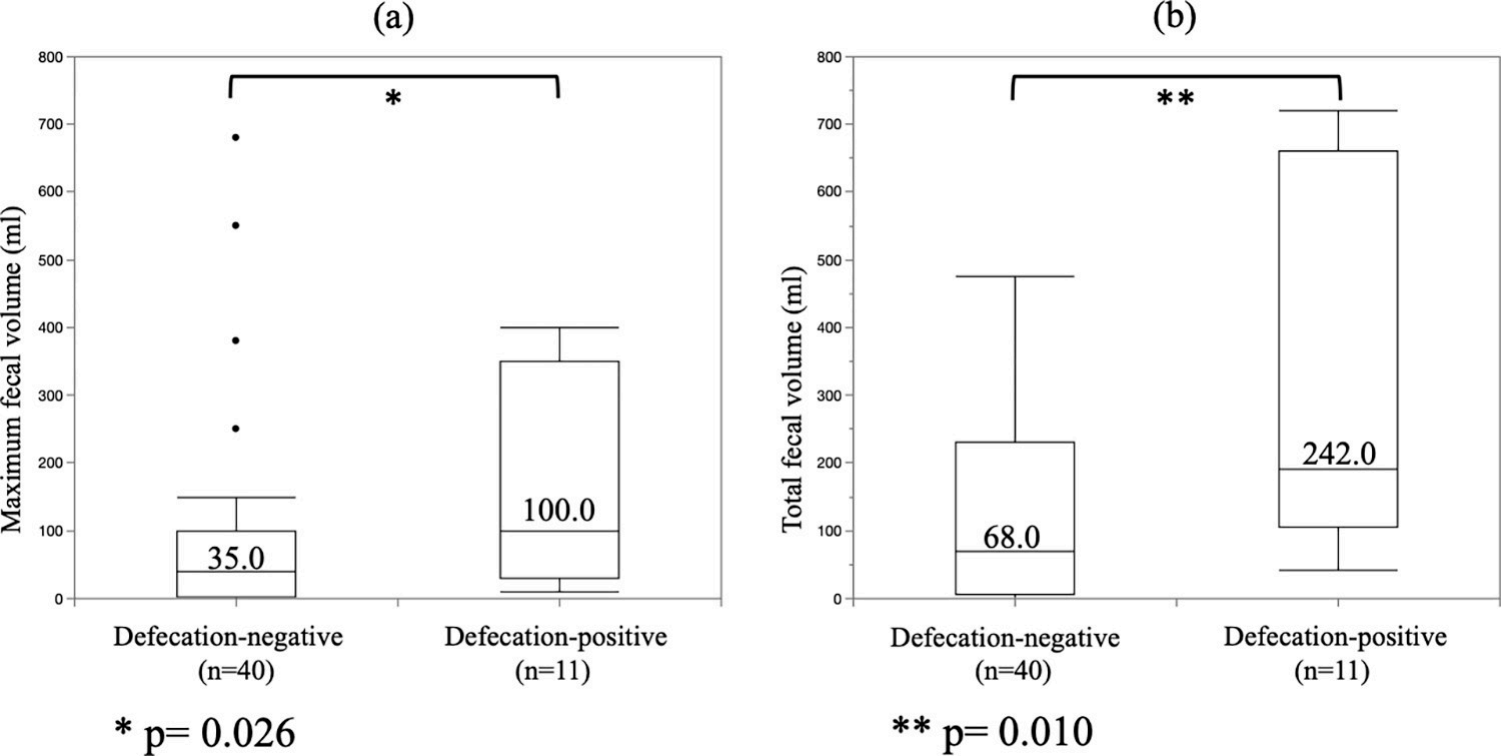
Association between intentional defecation and the fecal volume from the transanal drainage tube. (a) The defecation-positive group have a significantly greater maximum daily fecal volume during postoperative days 1–5 in comparison to the defecation-negative group (Median total fecal volume: 100.0 ml vs. 35.0 ml, respectively. p = 0.026). (b) The defecation-positive group have a significantly greater total fecal volume during postoperative days 1–5 in comparison to the defecation-negative group (Median total fecal volume: 242.0 ml vs. 68.0 ml, respectively. p = 0.010).

The median maximum fecal volume from the TDT during POD 1–5 in patients who experienced intentional defecation during TDT placement was 100.0ml (range 10.0 to 400.0; interquartile range 30.0 to 350.0). The median maximum fecal volume from the TDT during POD 1–5 in patients without intentional defecation during TDT placement was 35.0ml (range 0 to 680.0; interquartile range 2.3 to 95.0). The maximum fecal volume from the TDT during POD 1–5 in patients who experienced intentional defecation during TDT placement was significantly higher than that of patients without intentional defecation during TDT placement (p = 0.026) (Fig 2A). The median total fecal volume from the TDT during POD 1–5 in patients who experienced intentional defecation during TDT placement was 242.0ml (range 42.0 to 720.0; interquartile range 120.0 to 655.0). The median total fecal volume from the TDT during POD 1–5 in patients without intentional defecation during TDT placement was 68.0ml (range 0 to 1970.0; interquartile range 6.0 to 215.0). The total fecal volume from the TDT during POD 1–5 in patients who experienced intentional defecation during TDT placement was significantly higher than that of patients without intentional defecation during TDT placement (p = 0.010) (Fig 2B).

### The evaluation of risk factors of anastomotic leakage during TDT placement

The correlation between anastomotic leakage during TDT placement and risk factors was evaluated. In the univariate analysis, the anastomotic leakage rate during TDT placement in the group with a tumor diameter ≥35 mm and the group with a total fecal volume from TDT ≥260 ml during the first 5 postoperative days was significantly higher than in the group with a tumor diameter <35 mm and the group with a total fecal volume from TDT <260 ml (p = 0.006 and p = 0.0004, respectively). The multivariate analysis indicated that a large tumor diameter and total fecal volume from TDT during the first 5 postoperative days were independent risk factors for anastomotic leakage during TDT placement (p = 0.041 and p = 0.002, respectively) (Table 5).

**Table 5:** Univariate and multivariate analysis of risk factors of anastomotic leakage during TDT placement.

	univariate analysis			multivariate analysis		
	HR	95% CI	p-value	HR	95% CI	p-value
Sex (male vs. female)	5.813	0.559–60.47	0.109			
Age (<70 vs. ≥70)	2.875	0.279–29.68	0.345			
BMI (<25kg/m^2^ vs. ≥25kg/m^2^)	0.537	0.052–5.566	0.587			
Diameter of tumor (<35 mm vs. ≥35 mm)	8.901×10^7^	Not evaluable	0.006	2.0×10^8^	Not evaluable	0.041
The distance from anal marge (HAR vs. LAR)	4.05	0.392–41.87	0.206			
Total fecal volume from TDT during postoperative 5 days (<260 ml vs. ≥260 ml)	1.924×10^8^	Not evaluable	0.0004	5.599×10^8^	Not evaluable	0.002

HR: hazard ratio, CI: confidence interval, BMI: body mass index, HAR: high anterior resection, LAR: low anterior resection, TDT: transanal drainage tube

### Preoperative factors that were associated with the postoperative fecal volume from TDT

The total fecal volume from the TDT during POD 1–5 in patients in whom the tumor diameter was ≥35.0 mm tended to be higher than that of the patients in whom the tumor diameter was <35.0 mm (p = 0.051) (Table 6).

**Table 6:** Association between preoperative factors and the postoperative fecal volume from the transanal drainage tube.

Preoperative factor	Maximum daily fecal volume from TDT during POD1 to 5			Total fecal volume from TDT during POD1 to 5		
	<100.0 ml (n = 35)	≥100.0 ml (n = 16)	p-value	<260.0 ml (n = 39)	≥260.0 ml (n = 12)	p-value
Gender, n (%)						
Male	22 (62.9%)	10 (62.5%)	>0.999	24 (61.5%)	8 (66.7%)	0.872
Female	13 (37.1%)	6 (37.5%)		15 (38.5%)	4 (33.3%)	
Age (years)	69 (41–87)	72 (47–81)	0.684	70 (41–87)	70 (51–77)	0.601
Median (range)						
Operative method, n(%)						
High-anterior resection	20 (57.1%)	8 (50.0%)	0.764	23 (41.0%)	5 (41.7%)	0.292
Low-anterior resection	15 (42.9%)	8 (50.0%)		16 (59.0%)	7 (58.3%)	
Diameter of tumor, n (%)						
<35.0 mm	22 (62.9%)	8 (50.0%)	0.541	26 (66.7%)	4 (33.3%)	0.051
≥35.0 mm	13 (37.1%)	8 (50.0%)		13 (33.3%)	8 (66.7%)	
Surgical approach, n (%)						
Laparoscopic surgery	32 (91.4%)	16 (100%)	0.543	36 (92.3%)	12 (100%)	>0.999
Open surgery	3 (8.6%)	0 (0%)		3 (7.7%)	0 (0%)	

TDT: Transanal drainage tube; POD: postoperative day

### The subgroup analysis of the anastomotic leakage that occurred after TDT removal, among the patients who did not develop anastomotic leakage during TDT placement

Among the 47 patients who did not develop anastomotic leakage during TDT placement, 4 patients developed anastomotic leakage after removal of the TDT.

We used the daily fecal volume from the TDT on POD 5, which was a continuous variable, as the test variable and the occurrence of anastomotic leakage after removal of the TDT as the state variable. When we investigated the cut-off value for the daily fecal volume from the TDT on POD 5 using the ROC curve, we found that the appropriate cut-off value for the daily fecal volume from the TDT on POD 5 was 80.0 ml (sensitivity of 50.0%; specificity of 86.0%) (S4 Fig). We therefore set this fecal volume as the cut-off value and classified patients into high and low groups.

The anastomotic leakage rate after removal of the TDT of patients for whom the daily fecal volume from the TDT on POD 5 was ≥80.0 ml tended to be higher in comparison to the patients for whom the daily fecal volume from the TDT on POD 5 was <80.0 ml (p = 0.067) (Table 7).

**Table 7:** In the subgroup of 47 patients in whom no anastomotic leakage occurred during TDT placement, the association between the fecal volume from the transanal drainage tube on postoperative day 5 and the anastomotic leakage after removal of the transanal drainage tube.

	Anastomotic leakage after removal of TDT		p-value
	Negative (n = 43)	Positive (n = 4)	
Daily fecal volume from TDT of POD5, n(%)			
≥80.0 ml	6 (14.0%)	2 (50.0%)	0.067
<80.0 ml	37 (86.0%)	2 (50.0%)	

TDT: Transanal drainage tube; POD: Postoperative day

## Discussion

For many years, various methods have been adopted to prevent anastomotic leakage after surgery for rectal cancer. At our hospital, we confirm during surgery that the anastomotic site was not strained by noting sufficient descending colon mobilization. As needed, we add splenic flexura mobilization. We also confirm that sufficient blood flow is maintained to the distal resection margin. However, despite such management, the issue of anastomotic leakage remains.

TDT placement is as an easy and economical method for decompressing anastomosis. TDT was shown to be associated with patient discomfort [24] and a risk of bowel perforation [25, 26]. However, some reports have revealed that TDT placement reduces the rate of anastomotic leakage after resection of rectal cancer [27], and TDT placement for such reasons has now become popular. In addition, TDT was expected to reduce the discharge of feces into the abdominal cavity when anastomotic leakage occurred. TDT also has an advantage in that anastomotic leakage can be efficiently treated using both a TDT and an abdominal drainage tube when conservative treatment is performed [27].

Watery stool in the early period after surgery for rectal cancer has been reported to be a risk factor for anastomotic leakage [28–31]. However, there have been few reports on this topic and the relationship between the fecal volume after surgery and anastomotic leakage has remained unclear. In recent years, the TDT placement after surgery for rectal cancer has been widely performed, which has enabled the fecal volume from TDTs to be analyzed in detail. This has revealed an association between anastomotic leakage and the fecal volume from the TDT. The present study revealed that an increased fecal volume from the TDT was significantly associated with an increased rate of anastomotic leakage. These findings were in accordance with the results of previous studies, which found that the anastomotic leakage was associated with the fecal volume from the TDT after laparoscopic LAR [20, 29]. In our multivariate analysis, a large fecal volume from the TDT was an independent risk factor for anastomotic leakage during TDT placement. Based on the present findings in addition to the previously reported preoperative risk factors of anastomotic leakage, such as the tumor diameter or distance from the anal verge to the tumor, a large fecal volume from TDT was considered an important risk factor of anastomotic leakage.

In clinical practice, the defecation of watery stool which does not pass through the TDT may sometimes occur in spite of TDT placement after surgery. We therefore evaluated the correlation between defecation occurring during TDT placement and anastomotic leakage/fecal volume from the TDT. Our study revealed that intentional defecation during TDT placement was significantly associated with an increased anastomotic leakage rate. In addition, patients with intentional defecation during TDT placement had a significantly greater fecal volume after surgery than patients without intentional defecation during TDT placement. Given these results, one of the mechanisms underlying anastomotic leakage during TDT placement was suggested to involve a large volume of watery stool that occurred after surgery causing a large fecal volume from the TDT. Furthermore, when the volume of watery stool was larger than the volume that could flow through the TDT, it caused poor drainage from the rectum, and the remaining fecal matter in the rectum increased the risk of intentional defecation. With the intestinal pressure increasing many times due to intentional defecation, the substantial physical burden occurring at the site of anastomosis would then cause anastomosis rupture.

Several preoperative factors were expected to be related to the presence of watery stool in the early period after surgery increasing the fecal volume from the TDT. In our study, a tumor diameter ≥35 mm was suggested to be a risk factor for an increased fecal volume from the TDT after surgery. This may be because a large tumor can cause stenosis of the bowel, resulting in bowel preparation not providing sufficient elimination of the intestinal contents and contents therefore being left in the bowel after laxative administration. In addition, a large postoperative fecal volume was considered to occur due to a large amount of watery stool being moved into the rectum upon the release of the stenosis by surgery and the restart of intestinal peristalsis.

Given the above findings, we suggested that appropriate perioperative management for TDT placement was important for ensuring decompression in order to prevent anastomotic leakage during TDT placement. In cases with a large fecal volume from the TDT or intentional defecation, perioperative management should be strictly performed. It is necessary to pay attention not only to instances of fever flare or abdominal pain but also obstruction of the TDT due to bending and unintentional removal of the TDT. Furthermore, adequate bowel preparation should be devised in order to prevent severe watery diarrhea after surgery. Cases with a large tumor diameter in particular were regarded as high-risk cases for a large fecal volume, even if they had no symptom of ileus. In these cases, preoperative treatment, such as dietary restrictions or gradual laxative administration, should be performed to ensure the complete elimination of the intestinal contents.

On the other hand, there was no association between fecal incontinence during TDT placement after surgery and anastomotic leakage. Thus, fecal incontinence during TDT placement was considered to reflect good drainage of the rectum. Thus, the occurrence of fecal incontinence during TDT placement was not associated with a need for increased vigilance.

In the present study, some patients developed anastomotic leakage after the removal of the TDT. These patients developed anastomotic leakage after POD 5, likely due to postoperative factors, such as watery diarrhea. In these cases, the TDT was removed despite drainage by the TDT still being necessary, so frequent defecation occurred after its removal, and anastomotic leakage then developed due to the physical compression of the anastomotic site. Thus, in cases involving a high fecal volume on POD 5, it was suggested that treating the watery diarrhea and delaying the removal of the TDT or the start of meal intake might help prevent anastomotic leakage after the removal of the TDT.

The present study was associated with some limitations. First, this study was a retrospective study that included a relatively small number of patients who were managed at a single institution. As the number of the patients was quite small for statistical analyses, the analysis results may have low reproducibility. Second, the method of bowel preparation and the criteria for removal of the TDT were not uniform and they depended on the choice of each surgeon. Thus, a prospective study should be performed after establishing appropriate criteria, such as the methods of bowel preparation or the timing of removal of the TDT. Third, in Japan, neoadjuvant chemoradiotherapy for locally advanced rectal cancer is not a standard treatment strategy. Considering the effect of radiotherapy at the rectal anastomosis site, patients who had received neoadjuvant chemoradiotherapy were excluded from the present study. When more cases are accumulated, it will be necessary to evaluate the significance of the fecal volume from the TDT in patients who have undergone neoadjuvant chemotherapy as well.

## Conclusion

A large fecal volume from the TDT after anterior rectal resection or intentional defecation in patients with TDT placement were suggested to be risk factors for anastomotic leakage. To reduce the rate of anastomotic leakage, it is necessary to perform appropriate bowel preparation and thus reduce the postoperative fecal volume and to provide TDT management for appropriate drainage in the rectum.

## Supporting information

S1 Fig Association between anastomotic leakage and the diameter of tumor. The anastomotic leakage positive group have a significantly longer diameter in comparison to the anastomotic leakage negative group (Median diameter of tumor: 28.0mm; 55.0mm. p = 0.021).
(TIF)

S2 Fig The receiver operating characteristic curve of the diameter of tumor for anastomotic leakage. The receiver operating characteristic curve of the diameter of tumor for anastomotic leakage is shown. Area under the curve = 0.760; 95% confidence interval = 0.549–0.892; p = 0.088.
(TIF)

S3 Fig The receiver operating characteristic curve of the fecal volume from the transanal drainage tube from postoperative days 1–5 for anastomotic leakage during transanal drainage tube placement. (a) The receiver operating characteristic curve of the maximum daily fecal volume from the transanal drainage tube from postoperative days 1–5 for anastomotic leakage during transanal drainage tube placement is shown. Area under the curve = 0.891; 95% confidence interval = 0.741–0.959; p = 0.054. (b) Receiver operating characteristic curve of the total fecal volume from the transanal drainage tube from postoperative days 1–5 for anastomotic leakage during transanal drainage tube placement is shown. Area under the curve = 0.894; 95% confidence interval = 0.764–0.956; p = 0.152.
(TIF)

S4 Fig The receiver operating characteristic curve of the fecal volume from the transanal drainage tube on postoperative day 5 for anastomotic leakage after removal of the transanal drainage tube in the subgroup who did not develop anastomotic leakage during TDT placement. The receiver operating characteristic curve of the fecal volume from the transanal drainage tube on postoperative day 5 for anastomotic leakage after removal of the transanal drainage tube in the subgroup who did not develop anastomotic leakage during TDT placement is shown. Area under the curve = 0.637; 95% confidence interval = 0.267–0.894; p = 0.545.
(TIF)
