# Supplementary figures and images for: Significance of information obtained during transanal drainage tube placement after anterior resection of colorectal cancer
Source: PLoS One. 2022 Aug 29;17(8):e0271496. doi: 10.1371/journal.pone.0271496 (PMC9423657; doi:10.1371/journal.pone.0271496)

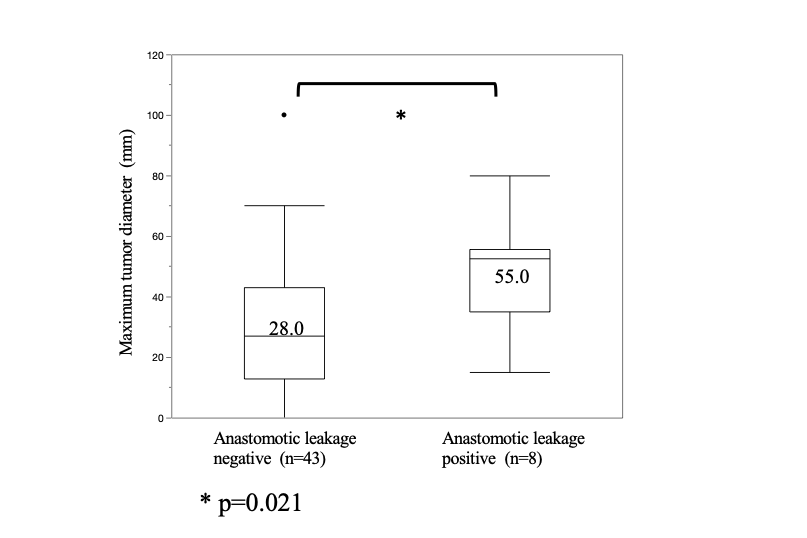

Supplement: S1 Fig — The anastomotic leakage positive group have a significantly longer diameter in comparison to the anastomotic leakage negative group (Median diameter of tumor: 28.0mm; 55.0mm. p = 0.021). (TIF) [file pone.0271496.s001.tif]

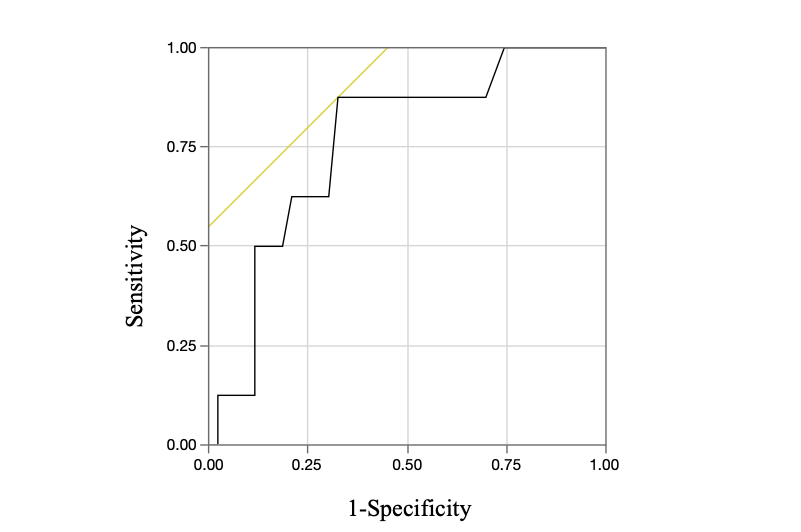

Supplement: S2 Fig — The receiver operating characteristic curve of the diameter of tumor for anastomotic leakage is shown. Area under the curve = 0.760; 95% confidence interval = 0.549–0.892; p = 0.088. (TIF) [file pone.0271496.s002.tif]

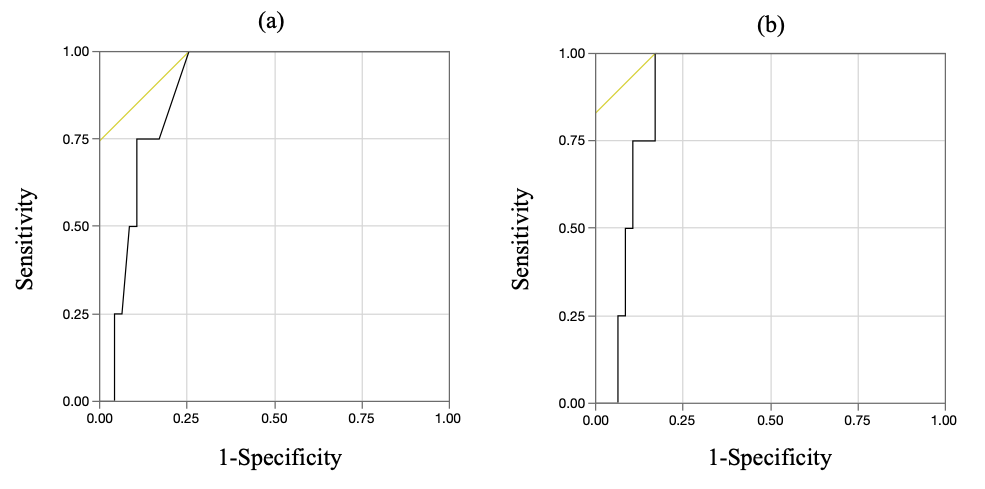

Supplement: S3 Fig — (a) The receiver operating characteristic curve of the maximum daily fecal volume from the transanal drainage tube from postoperative days 1–5 for anastomotic leakage during transanal drainage tube placement is shown. Area under the curve = 0.891; 95% confidence interval = 0.741–0.959; p = 0.054. (b) Receiver operating characteristic curve of the total fecal volume from the transanal drainage tube from postoperative days 1–5 for anastomotic leakage during transanal drainage tube placement is shown. Area under the curve = 0.894; 95% confidence interval = 0.764–0.956; p = 0.152. (TIF) [file pone.0271496.s003.tif]

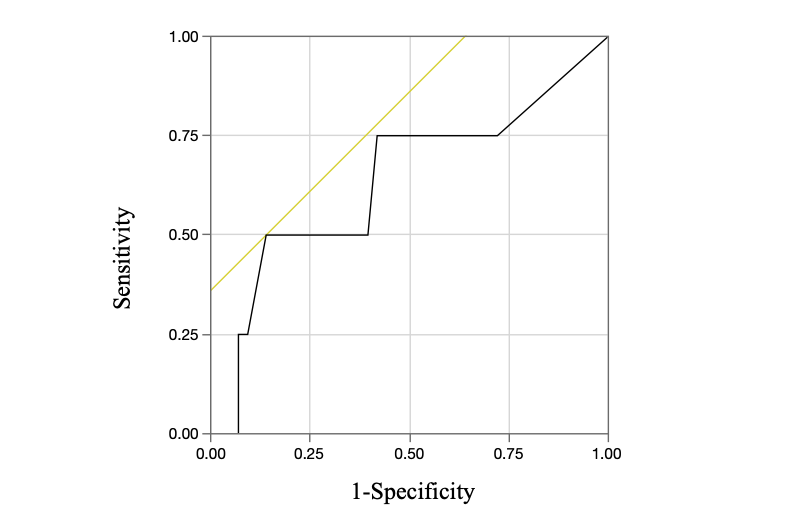

Supplement: S4 Fig — The receiver operating characteristic curve of the fecal volume from the transanal drainage tube on postoperative day 5 for anastomotic leakage after removal of the transanal drainage tube in the subgroup who did not develop anastomotic leakage during TDT placement is shown. Area under the curve = 0.637; 95% confidence interval = 0.267–0.894; p = 0.545. (TIF) [file pone.0271496.s004.tif]
