# Supplementary material for: Significance of information obtained during transanal drainage tube placement after anterior resection of colorectal cancer
Source: PLoS One. 2022 Aug 29;17(8):e0271496. doi: 10.1371/journal.pone.0271496 (PMC9423657; doi:10.1371/journal.pone.0271496)
Supplement: Attachment — Submitted filename: PONE-D-20-36803_reviewer.pdf [file pone.0271496.s005.pdf]

PLOS ONE

Significance of information obtained during transanal drainage tube placement after anterior resection of colorectal cancer

--Manuscript Draft--

**Manuscript Number:**	PONE-D-20-36803
**Article Type:**	Research Article
**Full Title:**	Significance of information obtained during transanal drainage tube placement after anterior resection of colorectal cancer
**Short Title:**	The information obtained during transanal drainage tube placement
**Corresponding Author:**	Masatsune Shibutani<br>Osaka City University Graduate School of Medicine<br>Osaka, JAPAN
**Keywords:**	transanal drainage tube; rectal cancer surgery; anastomotic leakage
**Abstract:**	Introduction<br><br>It has recently been reported that the placement of a transanal drainage tube after rectal cancer surgery reduces the rate of anastomotic leakage. However, transanal drainage tube cannot completely prevent anastomotic leakage and the management of transanal drainage tube needs to devise. We investigated the information obtained during transanal drainage tube placement and evaluated the relationship between these factors and anastomotic leakage.<br><br>Patients and Methods<br><br>Fifty-one patients who underwent anterior resection of rectal cancer was retrospectively reviewed. transanal drainage tube was placed for more than 5 days after surgery. The daily fecal volume from transanal drainage tube was measured on postoperative day 1-5, and the defecation during transanal drainage tube placement was investigated.<br><br>Results<br><br>Anastomotic leakage during transanal drainage tube placement occurred in 4 patients. The anastomotic leakage rate during transanal drainage tube placement of patients whose maximum daily fecal volume or total fecal volume from the transanal drainage tube during postoperative day 1-5 was large was significantly higher than that in patients whose fecal volume was small. The anastomotic leakage rate of the patients with intentional defecation during transanal drainage tube placement was significantly higher than that of the patients without intentional defecation during transanal drainage tube placement. The maximum daily fecal volume and the total fecal volume from the transanal drainage tube during postoperative day 1-5 in patients who experienced intentional defecation during transanal drainage tube placement was significantly higher than that of patients without intentional defecation during transanal drainage tube placement.<br><br>Conclusion<br><br>A large fecal volume from transanal drainage tube after anterior rectal resection or intentional defecation in patients with transanal drainage tube placement were suggested to be risk factors for anastomotic leakage.
**Order of Authors:**	Yuki Okazaki
	Masatsune Shibutani
	Hisashi Nagahara
	Tatsunari Fukuoka
	Yasuhito Iseki

Powered by Editorial Manager® and ProduXion Manager® from Aries Systems Corporation

	En Wang
	Kiyoshi Maeda
	Kosei Hirakawa
	Masaichi Ohira
**Additional Information:**	
**Question**	**Response**
**Financial Disclosure**<br><br>Enter a financial disclosure statement that describes the sources of funding for the work included in this submission. Review the submission guidelines for detailed requirements. View published research articles from *PLOS ONE* for specific examples.<br><br>This statement is required for submission and **will appear in the published article** if the submission is accepted. Please make sure it is accurate.<br><br>**Unfunded studies**<br>Enter: *The author(s) received no specific funding for this work.*<br><br>**Funded studies**<br>Enter a statement with the following details:<br>• Initials of the authors who received each award<br>• Grant numbers awarded to each author<br>• The full name of each funder<br>• URL of each funder website<br>• Did the sponsors or funders play any role in the study design, data collection and analysis, decision to publish, or preparation of the manuscript?<br>• **NO** - Include this sentence at the end of your statement: *The funders had no role in study design, data collection and analysis, decision to publish, or preparation of the manuscript.*<br>• **YES** - Specify the role(s) played.<br><br>* typeset	The authors received no specific funding for this work.
**Competing Interests**<br><br>Use the instructions below to enter a competing interest statement for this submission. On behalf of all authors, disclose any competing interests that	The authors have declared that no competing interests exist.

could be perceived to bias this work—acknowledging all financial support and any other relevant financial or non-financial competing interests.<br><br>This statement **will appear in the published article** if the submission is accepted. Please make sure it is accurate. View published research articles from *PLOS ONE* for specific examples.<br><br>**NO authors have competing interests**<br><br>Enter: *The authors have declared that no competing interests exist.*<br><br>**Authors with competing interests**<br><br>Enter competing interest details beginning with this statement:<br><br>*I have read the journal's policy and the authors of this manuscript have the following competing interests: [insert competing interests here]*<br><br>* typeset	
**Ethics Statement**<br><br>Enter an ethics statement for this submission. This statement is required if the study involved:<br><br>• Human participants<br>• Human specimens or tissue<br>• Vertebrate animals or cephalopods<br>• Vertebrate embryos or tissues<br>• Field research<br><br>Write "N/A" if the submission does not require an ethics statement.<br><br>General guidance is provided below. Consult the submission guidelines for detailed instructions. **Make sure that all information entered here is included in the Methods section of the manuscript.**	This study was approved by the Ethics Committee of Osaka City University (approval number: 4182) and conducted in accordance with the Declaration of Helsinki. All patients provided their written informed consent.

**Format for specific study types**<br><br>**Human Subject Research (involving human participants and/or tissue)**<br>• Give the name of the institutional review board or ethics committee that approved the study<br>• Include the approval number and/or a statement indicating approval of this research<br>• Indicate the form of consent obtained (written/oral) or the reason that consent was not obtained (e.g. the data were analyzed anonymously)<br><br>**Animal Research (involving vertebrate animals, embryos or tissues)**<br>• Provide the name of the Institutional Animal Care and Use Committee (IACUC) or other relevant ethics board that reviewed the study protocol, and indicate whether they approved this research or granted a formal waiver of ethical approval<br>• Include an approval number if one was obtained<br>• If the study involved *non-human primates*, add *additional details* about animal welfare and steps taken to ameliorate suffering<br>• If anesthesia, euthanasia, or any kind of animal sacrifice is part of the study, include briefly which substances and/or methods were applied<br><br>**Field Research**<br><br>Include the following details if this study involves the collection of plant, animal, or other materials from a natural setting:<br>• Field permit number<br>• Name of the institution or relevant body that granted permission	
**Data Availability**<br><br>Authors are required to make all data underlying the findings described fully available, without restriction, and from the time of publication. PLOS allows rare exceptions to address legal and ethical concerns. See the PLOS Data Policy and FAQ for detailed information.	Yes - all data are fully available without restriction

Powered by Editorial Manager® and ProduXion Manager® from Aries Systems Corporation

A Data Availability Statement describing where the data can be found is required at submission. Your answers to this question constitute the Data Availability Statement and **will be published in the article**, if accepted.<br><br>**Important:** Stating 'data available on request from the author' is not sufficient. If your data are only available upon request, select 'No' for the first question and explain your exceptional situation in the text box.<br><br>Do the authors confirm that all data underlying the findings described in their manuscript are fully available without restriction?	
**Describe where the data may be found in full sentences. If you are copying our sample text, replace any instances of XXX with the appropriate details.**<br><br>• If the data are **held or will be held in a public repository**, include URLs, accession numbers or DOIs. If this information will only be available after acceptance, indicate this by ticking the box below. For example: *All XXX files are available from the XXX database (accession number(s) XXX, XXX.)*.<br>• If the data are all contained **within the manuscript and/or Supporting Information files**, enter the following: *All relevant data are within the manuscript and its Supporting Information files.*<br>• If neither of these applies but you are able to provide **details of access elsewhere**, with or without limitations, please do so. For example:<br><br>*Data cannot be shared publicly because of [XXX]. Data are available from the XXX Institutional Data Access / Ethics Committee (contact via XXX) for researchers who meet the criteria for access to confidential data.*<br><br>*The data underlying the results presented in the study are available from (include the name of the third party*	All relevant data are within the manuscript and its Supporting Information files.

and contact information or URL).<br>• This text is appropriate if the data are owned by a third party and authors do not have permission to share the data.<br><br>* typeset	
Additional data availability information:	

Manuscript
Click here to access/download;Manuscript;PLOSONE manuscript OkazakiYuki.docx

Significance of information obtained during transanal drainage tube placement after anterior resection of colorectal cancer

Yuki Okazaki [1¶], Masatsune Shibutani [1*¶], Hisashi Nagahara [1¶], Tatsunari Fukuoka [1¶], Yasuhito Iseki [1¶], En Wang [1¶], Kiyoshi Maeda [2¶], Kosei Hirakawa [1¶], Masaichi Ohira [1¶]

[1] Department of Gastroenterological Surgery, Osaka City University Graduate School of Medicine, Abeno-ku, Osaka, Japan

[2] Department of Gastroenterological Surgery, Osaka City General Hospital, Miyakojima-ku, Osaka, Japan

[*]Corresponding author

E-mail: fbxbj429@ybb.ne.jp

[¶]These authors contributed equally to this work.

Short title: **Significance of information from TDT after anterior resection of colorectal cancer**

Conflicts of interest

The authors declare that they have no competing interests in regard to this study.

Abstract

Introduction: It has recently been reported that the placement of a transanal drainage tube after rectal cancer surgery reduces the rate of anastomotic leakage. However, transanal drainage tube cannot completely prevent anastomotic leakage and the management of transanal drainage tube needs to devise. We investigated the information obtained during transanal drainage tube placement and evaluated the relationship between these factors and anastomotic leakage. **Patients and Methods**: Fifty-one patients who underwent anterior resection of rectal cancer was retrospectively reviewed. transanal drainage tube was placed for more than 5 days after surgery. The daily fecal volume from transanal drainage tube was measured on postoperative day 1-5, and the defecation during transanal drainage tube placement was investigated. **Results**: Anastomotic leakage during transanal drainage tube placement occurred in 4 patients. The anastomotic leakage rate during transanal drainage tube placement of patients whose maximum daily fecal volume or total fecal volume from the transanal drainage tube during postoperative day 1-5 was large was significantly higher than that in patients whose fecal volume was small. The anastomotic leakage rate of the patients with intentional defecation during transanal drainage tube placement was significantly higher than that of the patients without intentional defecation during transanal drainage tube placement. The maximum daily

fecal volume and the total fecal volume from the transanal drainage tube during postoperative day 1-5 in patients who experienced intentional defecation during transanal drainage tube placement was significantly higher than that of patients without intentional defecation during transanal drainage tube placement. **Conclusion**: A large fecal volume from transanal drainage tube after anterior rectal resection or intentional defecation in patients with transanal drainage tube placement were suggested to be risk factors for anastomotic leakage.

Introduction

Anastomotic leakage after the resection of colorectal cancer is a serious complication that is associated with short-term outcomes, such as reoperation, extension of hospital stay, and increased perioperative mortality [1-5], as well as long-term oncological effects, such as a poor prognosis due to local recurrence [6-8]. The anastomotic leakage rate after resection of rectal cancer is higher in comparison to other colon cancers [9]. Thus, in order to prevent anastomotic leakage after low-anterior resection (LAR) of the rectum, various methods have been adapted, such as adequate mobilization of the colon [10], the use of intracorporeal reinforcing sutures [11] and evaluation of the blood flow by fluorescence imaging with indocyanine green (ICG) [12]. In addition, in cases in which there is considered to be a high risk of anastomotic leakage, such as cases with anastomosis at a low rectal position, diverting stomas can be constructed to reduce the burden of anastomosis[13]. Recently, it has been reported that the placement of a transanal drainage tube (TDT), which is technically easy and which can economically decompress the anastomotic site[14], is effective for preventing anastomotic leakage after rectal cancer surgery[15-17]. However, even if a TDT is used, defecation may occur that does not pass through the TDT, and anastomotic leakage may occur. Furthermore, the timing of the removal of the TDT was sometimes delayed based on the judgment of each surgeon.

Thus, there may be room for improving the method of managing TDT in the perioperative period. The present study therefore explored the mechanism underlying the occurrence of anastomotic leakage despite using a TDT by evaluating the association between the perioperative clinical information obtained during TDT placement and anastomotic leakage and suggested a strategy for preventing anastomotic leakage. Thus, we retrospectively evaluated the association between clinical information during TDT placement and anastomotic leakage.

Patients and Methods

Fifty-one consecutive patients underwent surgery for the treatment of colorectal cancer with the double staple technique (DST) and who underwent TDT placement for 5 days or more after surgery, at Osaka City University Hospital between January 2016 and March 2019. None of the 51 patients underwent construction of a diverting stoma. Patients treated with preoperative chemotherapy or chemoradiotherapy, and those who underwent decompression treatment for intestinal obstruction were excluded from the present study. The characteristics and clinical information of the 51 patients were retrospectively based on their electronic medical records. The associations between anastomotic leakage and preoperative risk factors for anastomotic leakage, such as male sex, anastomosis at a low position and large-diameter tumor[18-21] were evaluated. The World Health Organization has reported that the body mass index (BMI; weight / length^2) ≥25.0 kg/m^2 indicates an overweight status[22]. We therefore set 25.0 kg/m^2 as the cut-off value of the BMI. The patients were classified into High-anterior resection (HAR) group and LAR group. The cut-off value for the tumor diameter was calculated based on the receiver operating characteristic (ROC) curve.

The standard mechanical bowel preparation at our hospital was fasting after lunch and the internal use of polyethylene glycol solution at 14:00 on the day before surgery.

However, two patients had diarrhea before the mechanical bowel preparation. No patients received antibiotic prophylaxis. For all the patients, a 10-mm Pleats drain (Akita Sumitomo Bakelite, Japan) was inserted from the anus and positioned with the tip approximately 5 cm above the anastomotic site. The removal of the TDT was scheduled for postoperative day (POD) 5; however, removal was sometimes delayed depending on the judgment of each surgeon.

The daily fecal volume from TDT and the total fecal volume for the 5 days of TDT placement (PODs 1-5 after the resection for CRC) were measured.

The cut-off values of the fecal volume were calculated based on an ROC curve analysis in order to determine the relationship between anastomotic leakage and the fecal volume from the TDT. The patients were divided to two groups: the high volume group and the low volume group.

The fecal matter that did not pass through the TDT during POD 1–5 was investigated from electronic medical records. In this study, the defecation that patients consciously performed during TDT placement was defined as intentional defecation, while the discharge that flowed outside the TDT unconsciously was defined as fecal incontinence. We evaluated the associations between anastomotic leakage and intentional defecation and between anastomotic leakage and fecal incontinence.

In addition, a subgroup analysis of patients in whom no anastomotic leakage occurred during TDT placement was performed. The association between the fecal volume from the TDT at POD 5, when TDT removal was scheduled, and the anastomotic leakage after removal of TDT was evaluated.

The details of anastomotic leakage were collected from the medical records of each surgeon. Anastomotic leakage was defined by major leakage (e.g., fecal discharge from the abdominal drain tube or the discharging of contrast agent into the abdominal cavity during fluoroscopic examination) and by minor leakage (e.g., free air around the anastomotic site on CT after the patient presented fever or abdominal pain).

All of the statistical analyses were performed using JMP 14.2.0 (SAS Institute, Japan, Tokyo). The chi-squared test, the Fisher's exact test and Mann-Whitney U-test were used to analyze the significance of associations between 2 groups. P values of <0.05 were considered to indicate statistical significance.

This retrospective study was approved by the Ethics Committee of Osaka City University (approval number: 4182) and conducted in accordance with the Declaration of Helsinki. All patients provided their written informed consent.

Results

Patient characteristics

The patient characteristics are listed in Table 1. Of the 51 patients who were analyzed, 32 were male and 19 were female. The median age was 70 years (range: 41-87). The median BMI was 23.8 kg/m^2 (range: 15.4-33.5). LAR was performed for 23 patients and HAR was performed for 28 patients. Laparoscopic operations were performed for 48 patients and open surgery was performed for 3 patients. The median tumor diameter was 30.0 mm (range: 0-100.0).

Table 1. Patient characteristics

**Clinical factor**	**n＝51**
Gender, n(%)	32 (62.7%)
Male	19 (37.3%)
Female	
Age (years)	70 (41-87)
Median (range)	
BMI (kg/m^2)	23.8 (15.4-33.5)
Median (range)	
Operative method, n(%)	28 (54.9%)
High-anterior resection	23 (45.1%)
Low-anterior resection	
Surgical approach, n(%)	48 (94.1%)
Laparoscopic surgery	3 (5.9%)
Open surgery	

Diameter of tumor (mm)<br>Median (range)	30.0 (0-100.0)

BMI: Body Mass Index

Preoperative factors associated with anastomotic leakage.

We used the tumor diameter, which was a continuous variable, as the test variable and the occurrence of anastomotic leakage as the state variable. When we investigated the cut-off value for the tumor diameter using the ROC curve, we found that the appropriate cut-off value for the tumor diameter was 35.0 mm (sensitivity of 75.0%; specificity of 67.4%) (S1 Fig). We therefore set 35.0 mm as the cut-off value and classified patients into high and low groups based on this value.

The anastomotic leakage rate was significantly higher in the groups with LAR and ≥35.0 mm tumor diameter than in the other groups (p=0.016, p=0.006, respectively) (Table 2).

Table 2. Preoperative factors associated with anastomotic leakage.

Preoperative factor	Anastomotic leakage		p-valve
	Negative (n=43)	Positive (n=8)	
Gender, n(%)			
Male	27 (62.8%)	5 (62.5%)	>0.999
Female	16 (37.2%)	3 (37.5%)	
Age(years)	70	62.5	0.161
Median（range）	（41-87）	(51-80)	
BMI, n(%)			
$\geq$25.0kg/m^2	17 (39.5%)	2 (25.0%)	0.694
<25.0kg/m^2	26 (60.5%)	6 (75.0%)	
Operative method, n(%)			
High-anterior resection	27 (62.8%)	1 (12.5%)	0.016
Low-anterior resection	16 (37.2%)	7 (87.5%)	
Diameter of tumor, n(%)			
$\geq$35.0mm	14 (67.4%)	7 (87.5%)	0.006
<35.0mm	29 (32.6%)	1 (12.5%)	

BMI: Body Mass Index

Occurrence and timing of anastomotic leakage

Anastomotic leakage occurred in 8 patients (15.7%). Four of these 8 cases occurred during TDT placement, and 4 of the 8 cases occurred after the removal of the TDT.

Association between the fecal volume from the TDT during

POD 1-5 and the anastomotic leakage during TDT placement

We used the maximum daily fecal volume from the TDT during PODs 1-5, which was a continuous variable, as the test variable and the occurrence of anastomotic leakage during the TDT placement as the state variable. When we investigated the cut-off value for the maximum daily fecal volume from the TDT during PODs 1-5 using the ROC curve, we found that the appropriate cut-off value for the maximum daily fecal volume was 100.0 ml (sensitivity of 100.0%; specificity of 74.5%) (S2a Fig). Using the ROC curve in the same manner, we set the cut-off value for the total fecal volume from the TDT during PODs 1-5 at 260.0 ml (sensitivity of 100.0%; specificity of 83.0%) (S2b Fig). We therefore set each of these values of fecal volume as the relevant cut-off values and classified patients into the high and low groups.

The anastomotic leakage rate during TDT placement in patients in whom the maximum daily fecal volume from the TDT during POD 1-5 was ≥100.0 ml was significantly higher than that of the patients in whom the maximum daily fecal volume from the TDT during POD 1-5 was <100.0 ml ($p=0.007$). The anastomotic leakage rate during TDT placement in patients in whom the total fecal volume from the TDT during POD 1-5 was ≥260.0 ml was significantly higher than that of the patients in whom the total fecal volume from the TDT during POD 1-5 was <260.0 ml ($p=0.002$) (Table 3).

Table 3. Association between the fecal volume from the transanal drainage tube during postoperative day 1 to 5 and the anastomotic leakage during placement of transanal drainage tube.

	**Anastomotic leakage during TDT placement**		
	**Negative (n=47)**	**Positive (n=4)**	**p-valve**
Maximum daily fecal volume from TDT, n(%)			
≥100.0ml	12 (25.5%)	4 (100.0%)	0.007
<100.0ml	35 (74.5%)	0 (0%)	
Total fecal volume from TDT during POD1 to 5, n(%)			
≥260.0ml	8 (11.6%)	4 (100.0%)	0.002
<260.0ml	39 (88.4%)	0 (0%)	

TDT : Transanal drainage tube

Association between fecal discharge not through TDT and the anastomotic leakage during TDT placement.

The anastomotic leakage rate of the patients who experienced fecal incontinence during TDT placement was not significantly different from that in patients without fecal incontinence during TDT placement. However, the anastomotic leakage rate of patients who experienced intentional defecation during TDT placement was significantly higher than that of patients without intentional defecation during TDT placement (p=0.028)

(Table 4).

Table 4 Association between the anastomotic leakage during placement of transanal drainage tube and fecal discharge not through transanal drainage tube during transanal drainage tube placement.

	Anastomotic leakage during TDT placement		
	**Negative (n=47)**	**Positive (n=4)**	**p-valve**
Fecal incontinence, n(%)			
No	42 (89.4%)	4 (100.0%)	>0.999
Yes	5 (10.6%)	0 (0%)	
Intentional defecation, n(%)			
No	39 (83.0%)	1 (25.0%)	0.028
Yes	8 (17.0%)	3 (75.0%)	

TDT: transanal drainage tube; POD: postoperative day

Association between intentional defecation during TDT placement and the fecal volume from TDT.

The maximum fecal volume from the TDT during POD 1-5 in patients who

experienced intentional defecation during TDT placement was significantly higher than that of patients without intentional defecation during TDT placement (p=0.026) (Fig 1a). The total fecal volume from the TDT during POD 1-5 in patients who experienced intentional defecation during TDT placement was significantly higher than that of patients without intentional defecation during TDT placement (p=0.010) (Fig 1b).

Fig 1. Association between intentional defecation and the fecal volume from the transanal drainage tube.

The Fig 1. legend. (a) The defecation-positive group have a significantly greater maximum daily fecal volume during postoperative days 1–5 in comparison to the defecation-negative group (Median total fecal volume: 35.0 ml; 100.0 ml. p=0.026). (b) The defecation-positive group have a significantly greater total fecal volume during postoperative days 1–5 in comparison to the defecation-negative group (Median total fecal volume: 68.0 ml; 242.0 ml. p=0.010).

Preoperative factors that were associated with the postoperative fecal volume from TDT.

The total fecal volume from the TDT during POD 1-5 in patients in whom the tumor

diameter was ≥35.0 mm tended to be higher than that of the patients in whom the tumor diameter was <35.0 mm (p=0.051) (Table 5).

Table 5 Association between preoperative factors and the postoperative fecal volume from the transanal drainage tube.

Preoperative factor	Maximum daily fecal volume from TDT during POD1 to 5			Total fecal volume from TDT during POD1 to 5		
	**<100.0ml (n=35)**	**≥100.0ml (n=16)**	**p-valve**	**<260.0ml (n=39)**	**≥260.0ml (n=12)**	**p-valve**
Gender, n (%)						
Male	22 (62.9%)	10 (62.5%)	>0.999	24 (61.5%)	8 (66.7%)	0.872
Female	13 (37.1%)	6 (37.5%)		15 (38.5%)	4 (33.3%)	
Age (years) Median (range)	69 (41-87)	72 (47-81)	0.684	70 (41-87)	70 (51-77)	0.601
Operative method, n(%)						
High-anterior resection	20 (57.1%)	8 (50.0%)	0.764	23 (41.0%)	5 (41.7%)	0.292
Low-anterior resection	15 (42.9%)	8 (50.0%)		16 (59.0%)	7 (58.3%)	
Diameter of tumor, n (%)						
<35.0mm	22 (62.9%)	8 (50.0%)	0.541	26 (66.7%)	4 (33.3%)	0.051
≥35.0mm	13 (37.1%)	8 (50.0%)		13 (33.3%)	8 (66.7%)	
Surgical approach, n (%)						
Laparoscopic surgery	32 (91.4%)	16 (100%)	0.543	36 (92.3%)	12 (100%)	>0.999
Open surgery	3 (8.6%)	0 (0%)		3 (7.7%)	0 (0%)	

TDT: Transanal drainage tube; POD: postoperative day

The subgroup analysis of the anastomotic leakage that

occurred after TDT removal, among the patients who did not develop anastomotic leakage during TDT placement.

Among the 47 patients who did not develop anastomotic leakage during TDT placement, 4 patients developed anastomotic leakage after removal of the TDT.

We used the daily fecal volume from the TDT on POD 5, which was a continuous variable, as the test variable and the occurrence of anastomotic leakage after removal of the TDT as the state variable. When we investigated the cut-off value for the daily fecal volume from the TDT on POD 5 using the ROC curve, we found that the appropriate cut-off value for the daily fecal volume from the TDT on POD 5 was 80.0 ml (sensitivity of 50.0%; specificity of 86.0%) (S3 Fig). We therefore set this fecal volume as the cut-off value and classified patients into high and low groups.

The anastomotic leakage rate after removal of the TDT of patients for whom the daily fecal volume from the TDT on POD 5 was ≥80.0 ml tended to be higher in comparison to the patients for whom the daily fecal volume from the TDT on POD 5 was <80.0 ml ($p=0.067$) (Table 6).

Table 6 In the subgroup of 47 patients in whom no anastomotic leakage occurred during TDT placement, the association between the fecal volume from the transanal drainage tube on postoperative day 5 and the anastomotic leakage after removal of

the transanal drainage tube.

	Anastomotic leakage after removal of TDT		
	**Negative (n=43)**	**Positive (n=4)**	**p-valve**
Daily fecal volume from TDT of POD5, n(%)			
≥80.0ml	6 (14.0%)	2 (50.0%)	0.067
<80.0ml	37 (86.0%)	2 (50.0%)	

TDT: Transanal drainage tube; POD: Postoperative day

Discussion

Recently, TDT placement has become popular as an easy and economical method for decompressing anastomosis. However, TDT is associated with patient discomfort [23] and a risk of bowel perforation [24, 25]. It has been reported that TDT placement reduces the rate of anastomotic leakage after resection of rectal cancer [26]. TDT was also expected to reduce the discharge of feces into the abdominal cavity when anastomotic leakage occurred. In addition, TDT has an advantage in that anastomotic leakage can be efficiently treated using both a TDT and an abdominal drainage tube when conservative treatment is performed[26].

Watery stool in the early period after surgery for rectal cancer has been reported to be a risk factor for anastomotic leakage [27-30]. However, there have been few reports on this topic and the relationship between the fecal volume after surgery and anastomotic leakage has remained unclear. In recent years, the TDT placement after surgery for rectal cancer has been widely performed, which has enabled the fecal volume from TDTs to be analyzed in detail. This has revealed an association between anastomotic leakage and the fecal volume from the TDT [28]. Kawada et al. reported that the anastomotic leakage rate of patients in whom the daily fecal volume from the TDT after laparoscopic LAR

exceeded 100 ml/day for two or more days was significantly higher than that of those in whom the daily fecal volume from the TDT did not exceed 100 ml/day or in whom it only exceeded this cut-off value for one day [20]. In our study, it was similarly revealed that an increased fecal volume from the TDT was significantly associated with an increased rate of anastomotic leakage. Thus, the fecal volume from the TDT is considered to be useful for predicting anastomotic leakage.

Our study revealed that intentional defecation during TDT placement was significantly associated with an increased anastomotic leakage rate, and patients with intentional defecation during TDT placement have a significantly greater total fecal volume in the 5 days after surgery. Given these results, one of the mechanisms underlying ruptured anastomoses during TDT placement was suggested to be due to a large volume of watery stool that occurred after the operation, thus leading to poor drainage from the rectum and intentional defecation and eventually resulting in too much compression occurring at the site of anastomosis.

In addition, a larger tumor diameter was considered to be a risk factor for an increased fecal volume from the TDT after surgery. This may be because the large tumor caused stenosis of the bowel and preoperative laxative treatment did not provide sufficient elimination of the intestinal contents, causing a large amount of watery stool to be

excreted with the release of the stenosis.

Thus, the appropriate management of TDT is important for decompression to prevent anastomotic leakage during TDT placement. We should not only avoid bending the drainage tube to prevent obstruction but also devise a bowel preparation to prevent severe watery diarrhea after surgery. In particular, the present study suggested that appropriate bowel preparation was necessary for patients with a large tumor diameter.

In addition, intentional defecation during TDT placement was considered associated with a high risk of anastomotic leakage. In cases in which intentional defecation is observed, TDT management should be strictly performed. On the other hand, there was no association between fecal incontinence and anastomotic leakage. Thus, fecal incontinence was considered to reflect good drainage of the rectum and was not associated with a need for increased vigilance.

In the present study, some patients developed anastomotic leakage after the removal of the TDT. These patients developed anastomotic leakage after POD 5, likely due to postoperative factors, such as watery diarrhea. In these cases, the TDT was removed despite drainage by the TDT still being necessary, so frequent defecation occurred after its removal, and anastomotic leakage then developed due to the physical compression of the anastomotic site. Thus, in cases involving a high fecal volume on POD 5, it was

suggested that treating the watery diarrhea and delaying the removal of the TDT or the start of meal intake might help prevent anastomotic leakage after the removal of the TDT.

The present study was associated with some limitations. First, this study was a retrospective study that included a relatively small number of patients who were managed at a single institution. Second, the method of bowel preparation and the criteria for removal of the TDT were not uniform and they depended on the choice of each surgeon. Thus, a prospective study should be performed after establishing appropriate criteria, such as the methods of bowel preparation or the timing of removal of the TDT.

Conclusion

A large fecal volume from the TDT after anterior rectal resection or intentional defecation in patients with TDT placement were suggested to be risk factors for anastomotic leakage. To reduce the rate of anastomotic leakage, it is necessary to perform appropriate bowel preparation and thus reduce the postoperative fecal volume and to provide TDT management for appropriate drainage in the rectum.

Acknowledgments

This research received no specific grants from any funding agency in the public, commercial or not-for-profit sectors. We thank Mr. Brian Quinn for providing medical writing services on behalf of JMC.

References

1. Gessler B, Eriksson O, Angenete E. Diagnosis, treatment, and consequences of anastomotic leakage in colorectal surgery. Int J Colorectal Dis. 2017;32(4):549-56. Epub 2017/01/11. doi: 10.1007/s00384-016-2744-x. PubMed PMID: 28070659; PubMed Central PMCID: PMCPMC5355508.

2. Kube R, Mroczkowski P, Granowski D, Benedix F, Sahm M, Schmidt U, et al. Anastomotic leakage after colon cancer surgery: a predictor of significant morbidity and hospital mortality, and diminished tumour-free survival. Eur J Surg Oncol. 2010;36(2):120-4. Epub 2009/09/25. doi: 10.1016/j.ejso.2009.08.011. PubMed PMID: 19775850.

3. McArdle CS, McMillan DC, Hole DJ. Impact of anastomotic leakage on long-term survival of patients undergoing curative resection for colorectal cancer. Br J Surg. 2005;92(9):1150-4. Epub 2005/07/22. doi: 10.1002/bjs.5054. PubMed PMID: 16035134.

4. Thornton M, Joshi H, Vimalachandran C, Heath R, Carter P, Gur U, et al. Management and outcome of colorectal anastomotic leaks. Int J Colorectal Dis. 2011;26(3):313-20. Epub 2010/11/26. doi: 10.1007/s00384-010-1094-3. PubMed PMID: 21107847.

5. Chiu HC, Lin YC, Hsieh HM, Chen HP, Wang HL, Wang JY. The impact of complications on prolonged length of hospital stay after resection in colorectal cancer: A retrospective study of Taiwanese patients. J Int Med Res. 2017;45(2):691-705. Epub 2017/02/09. doi: 10.1177/0300060516684087. PubMed PMID: 28173723; PubMed Central PMCID: PMCPMC5536677.

6. Mirnezami A, Mirnezami R, Chandrakumaran K, Sasapu K, Sagar P, Finan P. Increased local recurrence and reduced survival from colorectal cancer following anastomotic leak: systematic review and meta-analysis. Ann Surg. 2011;253(5):890-9. Epub 2011/03/12. doi: 10.1097/SLA.0b013e3182128929. PubMed PMID: 21394013.

7. Branagan G, Finnis D. Prognosis after anastomotic leakage in colorectal surgery. Dis Colon Rectum. 2005;48(5):1021-6. Epub 2005/03/25. doi: 10.1007/s10350-004-0869-4. PubMed PMID: 15789125.

8. Ramphal W, Boeding JRE, Gobardhan PD, Rutten HJT, de Winter L, Crolla R, et al. Oncologic outcome and recurrence rate following anastomotic leakage after curative resection for colorectal cancer. Surg Oncol. 2018;27(4):730-6. Epub 2018/11/20. doi: 10.1016/j.suronc.2018.10.003. PubMed PMID: 30449500.

9. Matsuda M, Tsuruta M, Hasegawa H, Okabayashi K, Kondo T, Shimada T, et al. Transanal drainage tube placement to prevent anastomotic leakage following colorectal

cancer surgery with double stapling reconstruction. Surg Today. 2016;46(5):613-20. Epub 2015/08/02. doi: 10.1007/s00595-015-1230-3. PubMed PMID: 26231480.

10. Thomas MS, Margolin DA. Management of Colorectal Anastomotic Leak. Clin Colon Rectal Surg. 2016;29(2):138-44. Epub 2016/06/02. doi: 10.1055/s-0036-1580630. PubMed PMID: 27247539; PubMed Central PMCID: PMCPMC4882170.

11. Maeda K, Nagahara H, Shibutani M, Ohtani H, Sakurai K, Toyokawa T, et al. Efficacy of intracorporeal reinforcing sutures for anastomotic leakage after laparoscopic surgery for rectal cancer. Surg Endosc. 2015;29(12):3535-42. Epub 2015/02/13. doi: 10.1007/s00464-015-4104-2. PubMed PMID: 25673349.

12. Kawada K, Hasegawa S, Wada T, Takahashi R, Hisamori S, Hida K, et al. Evaluation of intestinal perfusion by ICG fluorescence imaging in laparoscopic colorectal surgery with DST anastomosis. Surg Endosc. 2017;31(3):1061-9. Epub 2016/06/29. doi: 10.1007/s00464-016-5064-x. PubMed PMID: 27351656.

13. Hamabe A, Ito M, Nishigori H, Nishizawa Y, Sasaki T. Preventive effect of diverting stoma on anastomotic leakage after laparoscopic low anterior resection with double stapling technique reconstruction applied based on risk stratification. Asian J Endosc Surg. 2018;11(3):220-6. Epub 2017/12/13. doi: 10.1111/ases.12439. PubMed PMID: 29230964.

14. Nishigori H, Ito M, Nishizawa Y. A novel transanal tube designed to prevent anastomotic leakage after rectal cancer surgery: the WING DRAIN. Surg Today. 2017;47(4):513-20. Epub 2016/08/11. doi: 10.1007/s00595-016-1392-7. PubMed PMID: 27506753.

15. Xiao L, Zhang WB, Jiang PC, Bu XF, Yan Q, Li H, et al. Can transanal tube placement after anterior resection for rectal carcinoma reduce anastomotic leakage rate? A single-institution prospective randomized study. World J Surg. 2011;35(6):1367-77. Epub 2011/03/26. doi: 10.1007/s00268-011-1053-3. PubMed PMID: 21437746.

16. Yang Y, Shu Y, Su F, Xia L, Duan B, Wu X. Prophylactic transanal decompression tube versus non-prophylactic transanal decompression tube for anastomotic leakage prevention in low anterior resection for rectal cancer: a meta-analysis. Surg Endosc. 2017;31(4):1513-23. Epub 2016/09/14. doi: 10.1007/s00464-016-5193-2. PubMed PMID: 27620910.

17. Shigeta K, Okabayashi K, Baba H, Hasegawa H, Tsuruta M, Yamafuji K, et al. A meta-analysis of the use of a transanal drainage tube to prevent anastomotic leakage after anterior resection by double-stapling technique for rectal cancer. Surg Endosc. 2016;30(2):543-50. Epub 2015/06/21. doi: 10.1007/s00464-015-4237-3. PubMed PMID: 26091985.

18. Schrock TR, Deveney CW, Dunphy JE. Factor contributing to leakage of colonic anastomoses. Ann Surg. 1973;177(5):513-8. Epub 1973/05/01. doi: 10.1097/00000658-197305000-00002. PubMed PMID: 4540874; PubMed Central PMCID: PMCPMC1355583.

19. Sparreboom CL, van Groningen JT, Lingsma HF, Wouters M, Menon AG, Kleinrensink GJ, et al. Different Risk Factors for Early and Late Colorectal Anastomotic Leakage in a Nationwide Audit. Dis Colon Rectum. 2018;61(11):1258-66. Epub 2018/09/22. doi: 10.1097/dcr.0000000000001202. PubMed PMID: 30239395.

20. Kawada K, Takahashi R, Hida K, Sakai Y. Impact of transanal drainage tube on anastomotic leakage after laparoscopic low anterior resection. Int J Colorectal Dis. 2018;33(3):337-40. Epub 2017/12/23. doi: 10.1007/s00384-017-2952-z. PubMed PMID: 29270785.

21. Law WI, Chu KW, Ho JW, Chan CW. Risk factors for anastomotic leakage after low anterior resection with total mesorectal excision. Am J Surg. 2000;179(2):92-6. Epub 2000/04/25. doi: 10.1016/s0002-9610(00)00252-x. PubMed PMID: 10773140.

22. Clinical guidelines on the identification, evaluation, and treatment of overweight and obesity in adults: executive summary. Expert Panel on the Identification, Evaluation, and Treatment of Overweight in Adults. Am J Clin Nutr. 1998;68(4):899-917. Epub

1998/10/15. doi: 10.1093/ajcn/68.4.899. PubMed PMID: 9771869.

23. Kawachi J, Kashiwagi H, Shimoyama R, Isogai N, Fukai R, Miyake K, et al. Comparison of efficacies of the self-expandable metallic stent versus transanal drainage tube and emergency surgery for malignant left-sided colon obstruction. Asian J Surg. 2018;41(5):498-505. Epub 2017/08/29. doi: 10.1016/j.asjsur.2017.06.003. PubMed PMID: 28844781.

24. Wang S, Zhang Z, Liu M, Li S, Jiang C. Efficacy of transanal tube placement after anterior resection for rectal cancer: a systematic review and meta-analysis. World J Surg Oncol. 2016;14:92. Epub 2016/04/01. doi: 10.1186/s12957-016-0854-0. PubMed PMID: 27030245; PubMed Central PMCID: PMCPMC4815125.

25. Nishigori H, Ito M, Nishizawa Y, Kobayashi A, Sugito M, Saito N. Effectiveness of a transanal tube for the prevention of anastomotic leakage after rectal cancer surgery. World J Surg. 2014;38(7):1843-51. Epub 2014/01/01. doi: 10.1007/s00268-013-2428-4. PubMed PMID: 24378550.

26. Shalaby M, Thabet W, Buonomo O, Lorenzo ND, Morshed M, Petrella G, et al. Transanal Tube Drainage as a Conservative Treatment for Anastomotic Leakage Following a Rectal Resection. Ann Coloproctol. 2018;34(6):317-21. Epub 2018/12/24. doi: 10.3393/ac.2017.10.18. PubMed PMID: 30572421; PubMed Central PMCID:

PMCPMC6347340.
27. Ito T, Obama K, Sato T, Matsuo K, Inoue H, Kubota K, et al. Usefulness of transanal tube placement for prevention of anastomotic leakage following laparoscopic low anterior resection. Asian J Endosc Surg. 2017;10(1):17-22. Epub 2016/07/28. doi: 10.1111/ases.12310. PubMed PMID: 27456989.
28. Hidaka E, Maeda C, Nakahara K, Shimada S, Mukai S, Sawada N, et al. Fecal Volume after Laparoscopic Low Anterior Resection Predicts Anastomotic Leakage. Dig Surg. 2017;34(5):394-9. Epub 2017/01/19. doi: 10.1159/000454960. PubMed PMID: 28099959.
29. Hiraki M, Tanaka T, Ikeda O, Sadashima E, Kimura N, Nakamura S, et al. Retrospective Risk Analysis for Anastomotic Leakage Following Laparoscopic Rectal Cancer Surgery in a Single Institute. J Gastrointest Cancer. 2020;51(3):908-13. Epub 2019/11/13. doi: 10.1007/s12029-019-00315-9. PubMed PMID: 31713046.
30. Alves A, Panis Y, Pocard M, Regimbeau JM, Valleur P. Management of anastomotic leakage after nondiverted large bowel resection. J Am Coll Surg. 1999;189(6):554-9. Epub 1999/12/10. doi: 10.1016/s1072-7515(99)00207-0. PubMed PMID: 10589591.

Supporting information

S1 Fig. The receiver operating characteristic curve of the diameter of tumor for anastomotic leakage.

The receiver operating characteristic curve of the diameter of tumor for anastomotic leakage is shown. Area under the curve=0.760; 95% confidence interval=0.549-0.892; p=0.088.

S2 Fig. The receiver operating characteristic curve of the fecal volume from the transanal drainage tube from postoperative days 1–5 for anastomotic leakage during transanal drainage tube placement.

(a) The receiver operating characteristic curve of the maximum daily fecal volume from the transanal drainage tube from postoperative days 1–5 for anastomotic leakage during transanal drainage tube placement is shown. Area under the curve=0.891; 95% confidence interval=0.741-0.959; p=0.054. (b) Receiver operating characteristic curve of the total fecal volume from the transanal drainage tube from postoperative days 1–5 for anastomotic leakage during transanal drainage tube placement is shown. Area under the curve=0.894; 95% confidence interval=0.764-0.956; p=0.152.

S3 Fig. The receiver operating characteristic curve of the fecal volume from the transanal drainage tube on postoperative day 5 for anastomotic leakage after removal of the transanal drainage tube in the subgroup who did not develop anastomotic leakage during TDT placement.

The receiver operating characteristic curve of the fecal volume from the transanal drainage tube on postoperative day 5 for anastomotic leakage after removal of the transanal drainage tube in the subgroup who did not develop anastomotic leakage during TDT placement is shown. Area under the curve=0.637; 95% confidence interval=0.267-0.894; p=0.545.

1

Figure Click here to access/download;Figure;PLOSONE Figure 1 OkazakiYuki.tiff

Fig 1

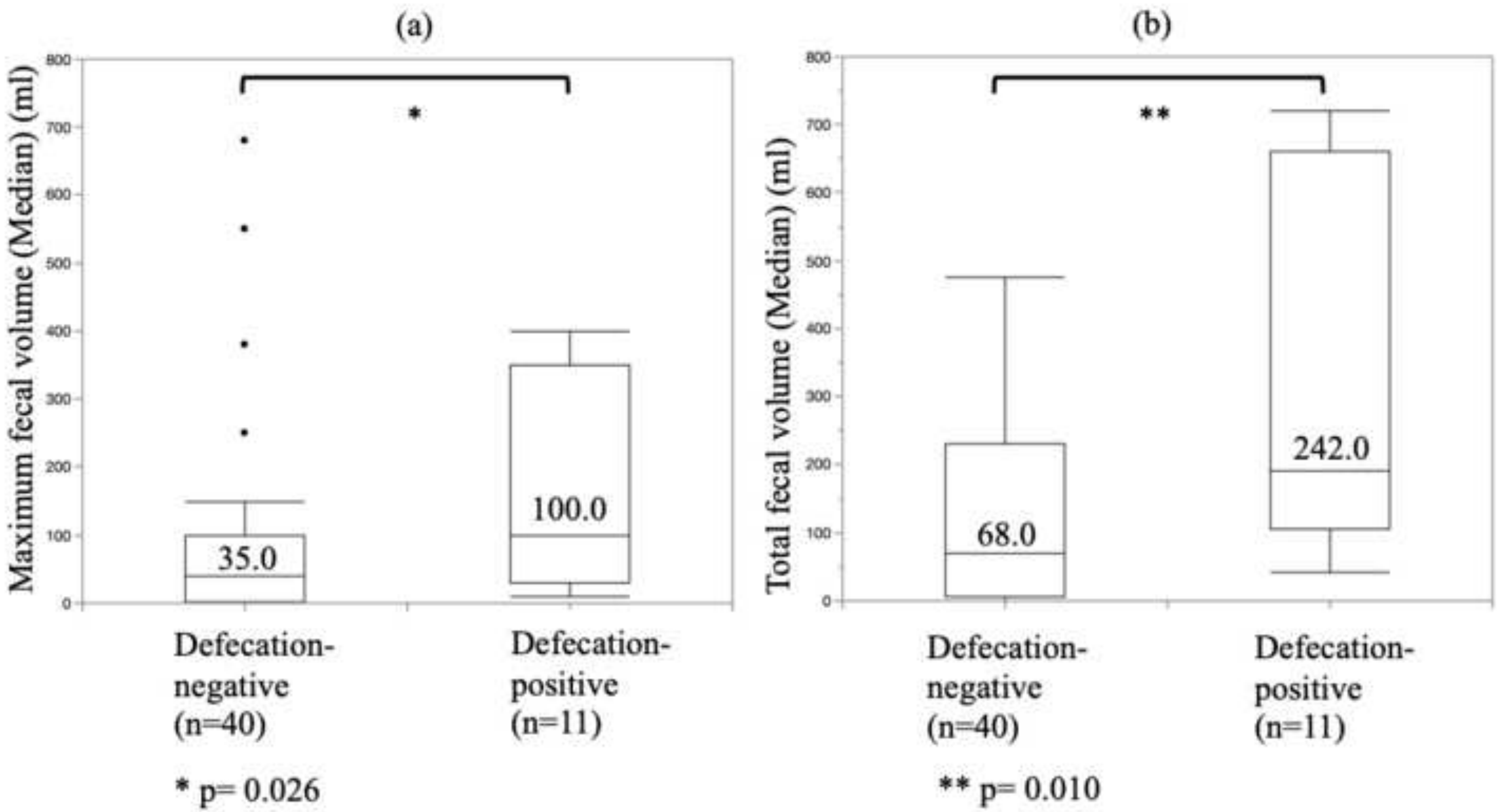

Click here to access/download
Supporting Information
renamed_0bd34.tiff
