# Supplementary material for: Significance of information obtained during transanal drainage tube placement after anterior resection of colorectal cancer
Source: PLoS One. 2022 Aug 29;17(8):e0271496. doi: 10.1371/journal.pone.0271496 (PMC9423657; doi:10.1371/journal.pone.0271496)
Supplement: Attachment — Submitted filename: Review-Plos One.docx [file pone.0271496.s006.docx]

MAJOR REVISION

The article describes a technically sound scientific research that will benefit in clinical practice. The manuscript is easy to understand and written in standard English. However, the results are not appropriately drawn based on the data presented. The results of the study was not strongly defended in the discussion. Therefore, my recommendations are below:

1. It is unclear how and where (operating room?, postoperatively in the clinic?, colonoscopically?) the pleats drain was inserted. It should be mentioned in more detail in the patients and methods section.
2. In this study, it was stated that TDT is effective in preventing anastomotic leakage. But, it would be more effective to compare the TDT group with a control group without TDT to asses whether it is effectual or not in preventing the leakage.
3. In the study, a single dependent group that received TDT for all cases was analyzed. However, analyzed subgroups do not have the clear definitons in the patients and methods section.
4. It was stated that cases were diveded into “high” and “low” subgroups according to tumor diameters estimated 35 mm cut-off value by the ROC analysis in the results section. Firstly, before calculating the cut-off value and creating subgroups, the relationship between tumor diameter and anastomotic leakage should be demonstrated by correlation tests. If a significant association between them was found, it would be more robust to give the cut-off value by ROC analysis.
5. In the present study involving a single dependent group, patients were divided into the other two subgroups as “high” and “low” according to cut-off value for the fecal volume drained from the TDT, and thus patients were distinguished according to the presence of anastomotic leakage. What should be done before determining a cut-off value and creating subgroups is to demonstrate a significant association between fecal volume and anastomotic leakage by using correlation tests.
6. Table 2 shows the preoperative factors associated with anastomotic leakage. What is the statistical method used here? Were preoperative parameters just compared? Or did multiple factors that may affect anastomotic leakage as the dependent variable analyze?
7. There seems to be a comparison of the anastomotic leakage with different defecation statuses in table 4, which is difficult to understand. According the table 4, why is the total number of patients without fecal incontinence (46 cases) not equal to the total number of patients with intentional defecation (11 cases)?
8. How many of patients had major and minor anastomotic leakage? What is the re-operation rate due to leakage?
9. All patients had the standard mechanical bowel preparation on the day before surgery. So, when did the first defecation postoperatively occur?
10. Leakage was reported in the other 4 of 51 patients after TDT was removed. However, there are again 47 patients in table 6. Where are the remaining 4 patients?
11. The discussion section has generally superficial content. The results of the study were not sufficiently interpreted and discussed. Especially, the association between tumor diameter and fecal volume and its mechanism lack the support of the literature. Therefore, the discussion section should be revised.
