# Supplementary material for: Significance of information obtained during transanal drainage tube placement after anterior resection of colorectal cancer
Source: PLoS One. 2022 Aug 29;17(8):e0271496. doi: 10.1371/journal.pone.0271496 (PMC9423657; doi:10.1371/journal.pone.0271496)
Supplement: Attachment — Submitted filename: Response to Reviewers.docx [file pone.0271496.s007.docx]

**Responses to the reviewer’s comments**

We wish to express our appreciation to the reviewers for their insightful comments, which have helped us significantly improve the paper.

**Reviewer 1**

Reviewer #1: Dear Author. Thanks for your article and precious work. I have some concerns about the methods using for measuring the fecal volume. I dont think if it is possible all fecal volume is passing through tdt. And also in some major studies and metaanalyses fecal volume is not one of the major risk factors. Splenic flexura mobilization, tension free anastamosis with a good blood supply must be the main purposes for a good anastomosis.

**Response:** As you pointed out, splenic flexura mobilization of the colon or tension-free anastomosis with a good blood supply is important for preventing anastomotic leakage. Therefore, in our facility, we mobilize the descending colon sufficiently and add mobilization of the splenic flexura as needed while confirming a good blood supply to the anastomosis by fluorescence imaging with indocyanine green. In the present study, we did not intend for TDT management to be the only method for preventing anastomotic leakage. We reported the significance of TDT management in addition to these already known methods to prevent anastomotic leakage.

In previous papers, postoperative diarrhea has been reported as a risk factor of anastomotic leakage. However, only a few reports have explored whether or not the fecal volume is a risk factor. In two meta-analyses, the fecal volume was not evaluated as a risk factor of anastomotic leakage, so whether or not the postoperative fecal volume is a major risk factor is unclear. TDT placement has facilitated the evaluation of the postoperative fecal volume in recent years. Therefore, we investigated whether or not the postoperative fecal volume through the TDT was a risk factor of anastomotic leakage.

The TDT was connected to the closed bag immediately after surgery, thus allowing the fecal volume through the TDT to be measured exactly. While measuring the fecal volume that did not pass through the TDT was difficult and the exact fecal volume in the rectum was not measured, we investigated the occurrence of intentional defecation or fecal incontinence as an alternative to determine the unmeasurable fecal volume. We evaluated the associations between anastomotic leakage and intentional defecation/fecal incontinence.

**Reviewer 2**

Q1. Page 7, line 4 Comment: 51 patients in 3 years means a low volume for colorectal surgery which is a risk factor for anastomotic leakage.

**Response:** As you pointed out, our total of 51 patients was quite small for the number of cases expected to be accumulated over 3 years. We considered it important to minimize the variation in the patient background. Therefore, the following patients were excluded from this study: those who underwent emergency operation, those who underwent NACRT, those who had ileus before surgery, those operated with an anastomosis method other than DST, those who received diverting stoma and those whose TDT was removed during PODs 1-4. After excluding the above patients, we were left with only 51 target patients, all of who showed little variation in their background.

Q2. Page 8, line 9: Did you have the data for the first gas passing in the postoperative period? If yes do you think adding this information may add value to your study?

**Response:** As the gas during TDT placement was basically drained through the TDT, the date of the first postoperative gas passage could not be determined. It is thus difficult to add these data to the manuscript.

Q3. Page 10, Line 6: 3 patients who had open surgery should be excluded from the study in order to perform a unique evaluation and a more proper statistal analyses.

**Response:** The fecal volume from the TDT did not markedly differ between laparoscopic and open surgery (P20 Table6 in the revised manuscript). In addition, had we excluded the two open surgery cases, the sample size in this study would have been even smaller. We therefore feel that these cases should not be excluded.

Q4. Page 11, Line 3: What was the preoperative grade of the tumors for the patients? Especially for the LAR group, why did not patients receieved neo-adjuvant chemo-radio therapy?

**Response:** We have now added the pathological T/N factors of the patients to Tables 1 and 2. The pathological T stage of 47 patients was T1-3, and that of 4 patients was T4. The pathological N stage of 43 patients was N0, and that of 8 patients was N1-3. The anastomotic leakage rate was not significantly higher in the groups with T4 or N1-3 than in the other groups. Please confirm the revised manuscript (P10-13 patient characteristics, Table 1 and 2 in the revised manuscript). We have also added the No23 reference.

In Japan, neoadjuvant chemoradiotherapy (NACRT) for locally advanced rectal cancer is not a standard treatment strategy. While NACRT is occasionally performed, we create diverting stomas in such cases. Patients who received NACRT were therefore excluded in this study. We have now mentioned this in the limitations section; please confirm the revised manuscript. (P27 lines11-16)

Q5. Table 2: LAR known to be more risky for the anastomotıc leakage. Why the high anterior group had more anastomotic leakage when compared to LAR?

**Response:** In the present study, the anastomotic leakage rate was higher in patients who underwent LAR than in those who underwent HAR. Please see Table 2 in our manuscript (P12).

Q6. Page 22, line 15: The authors should explain other possible rısk factors. Watery diarrhea should not be the only factor that is emphasized repeatedly.

**Response:** The correlations between the preoperative factors and anastomotic leakage in the present study are shown in Table 2, and the correlations between the risk factors of anastomotic leakage and fecal volume from the TDT are shown in Table 5 (P20 Table 6 in the revised manuscript). However, we did not present the results of the multivariate analysis for risk factors of the four anastomotic leakage incidents that occurred during TDT placement. The multivariate analysis indicated that a large tumor diameter and large total fecal volume from the TDT during the first 5 postoperative days were independent risk factors for anastomotic leakage during TDT placement. We have now mentioned these findings in the manuscript. Please see Table 5 in the revised manuscript (P19).

Cf. Table5. Univariate and multivariate analysis of risk factors of anastomotic leakage during TDT placement.

|  | **Univariate analysis** | | | **Multivariate analysis** | | |
| --- | --- | --- | --- | --- | --- | --- |
|  | **HR** | **95% CI** | **p-value** | **HR** | **95% CI** | **p-value** |
| Sex  (male vs. female) | 5.813 | 0.559-60.47 | 0.109 |  |  |  |
| Age  (<70 vs. ≥70 years old) | 2.875 | 0.279-29.68 | 0.345 |  |  |  |
| BMI  (<25 vs. ≥25 kg/m^2^) | 0.537 | 0.052-5.566 | 0.587 |  |  |  |
| Diameter of tumor  (<35 vs. ≥35 mm) | 8.901×10^7^ | Not evaluable | 0.006 | 2.0×10^8^ | Not evaluable | 0.041 |
| The distance from anal marge  (HAR vs. LAR) | 4.05 | 0.392-41.87 | 0.206 |  |  |  |
| Total fecal volume from TDT during the first 5 postoperative days  (<260 vs. ≥260 ml) | 1.924×10^8^ | Not evaluable | 0.0004 | 5.599×10^8^ | Not evaluable | 0.002 |
| BMI: body mass index, HAR: high anterior resection, LAR: low anterior resection, TDT: transanal drainage tube | | | | | | |

Q7. Page 23, Line 8: Authors should add a paragraph about the relation between BMI and anastomotic leakage to the discussion which is emphasized at the patients characteristics section.

**Response:** While a few reports have described the correlation between the BMI and anastomotic leakage, the mechanism underlying the correlation has been unclear, and the evidence level has been low. In the present study, no correlation between the BMI and anastomotic leakage was noted, so we omitted the details of this examination.

**Reviewer 3**

MAJOR REVISION

The article describes a technically sound scientific research that will benefit in clinical practice. The manuscript is easy to understand and written in standard English. However, the results are not appropriately drawn based on the data presented. The results of the study was not strongly defended in the discussion. Therefore, my additional recommendations are below:

Thank you for your constructive comment. We performed a deeper investigation and revised our manuscript as shown below.

Q1. It is unclear how and where (operating room?, postoperatively in the clinic?, colonoscopically?) the pleats drain was inserted. It should be mentioned in more detail in the patients and methods section.

**Response:** In our study, all of the TDTs were placed at the last step of the colorectal cancer operation under general anesthesia. We have now mentioned this in the Methods section (P8 lines8-9). Please confirm the revised manuscript.

Q2. In this study, it was stated that TDT is effective in preventing anastomotic leakage. But, it would be more effective to compare the TDT group with a control group without TDT to asses whether it is effectual or not in preventing the leakage.

**Response:** We began placing TDTs for all patients from January 2016. Before January 2016, TDT placement was only performed for patients considered to be at high risk of anastomotic leakage by the surgeon. In addition, the kind of TDT was not unified. Therefore, selection bias likely occurred, making it difficult to adequately prove the usefulness of a TDT for preventing anastomotic leakage based on an analysis of our hospital data.

Based on the premise outlined in previous reports that TDTs were effective for preventing anastomotic leakage after rectal cancer, we evaluated the correlation between anastomotic leakage and the fecal volume from the TDT.

**Response:** In the Methods section, we have defined a subgroup of patients in whom no anastomotic leakage occurred after TDT placement (P9 lines6-9). Please confirm the revised manuscript.

Q3. In the study, a single dependent group that received TDT for all cases was analyzed. However, analyzed subgroups do not have the clear definitons in the patients and methods section.

Q4. It was stated that cases were diveded into “high” and “low” subgroups according to tumor diameters estimated 35 mm cut-off value by the ROC analysis in the results section. Firstly, before calculating the cut-off value and creating subgroups, the relationship between tumor diameter and anastomotic leakage should be demonstrated by correlation tests. If a significant association between them was found, it would be more robust to give the cut-off value by ROC analysis.

**Response:** We have now added a new figure and explanation concerning the correlation between a large tumor diameter and anastomotic leakage before the ROC analysis. The tumor diameter in the patients who experienced anastomotic leakage was significantly greater than that in the patients without anastomotic leakage. Please confirm S1 Figure in the revised manuscript (P37).

Q5. In the present study involving a single dependent group, patients were divided into the other two subgroups as “high” and “low” according to cut-off value for the fecal volume drained from the TDT, and thus patients were distinguished according to the presence of anastomotic leakage. What should be done before determining a cut-off value and creating subgroups is to demonstrate a significant association between fecal volume and anastomotic leakage by using correlation tests.

**Response:** We have now added a new figure and explanation concerning the correlation between the fecal volume from the TDT and anastomotic leakage before the ROC analysis. The maximum fecal volume from the TDT during POD 1-5 in patients who experienced anastomotic leakage during TDT placement was significantly greater than that in patients without anastomotic leakage during TDT placement (Fig. 1a). The total fecal volume from the TDT during POD 1-5 in patients who experienced anastomotic leakage during TDT placement was significantly greater than that in patients without anastomotic leakage during TDT placement (Fig. 1b). Please confirm the revised manuscript (P15 lines 10-18).

Q6. Table 2 shows the preoperative factors associated with anastomotic leakage. What is the statistical method used here? Were preoperative parameters just compared? Or did multiple factors that may affect anastomotic leakage as the dependent variable analyze？

**Response:** Table 2 shows the difference in the background characteristics between the anastomotic leakage-positive group and the anastomotic leakage-negative group. However, this result alone was not enough to exclude confounding factors when risk factors for anastomotic leakage during TDT placement were evaluated.

Therefore, we performed a multivariate analysis of the risk factors of anastomotic leakage during TDT placement. We have now added a new table and appropriate explanation. The multivariate analysis indicated that a large tumor diameter and large total fecal volume from the TDT during the first 5 postoperative days were independent risk factors for anastomotic leakage during TDT placement (P19 Table 5 in the revised manuscript). Please confirm the revised manuscript.

Q7. There seems to be a comparison of the anastomotic leakage with different defecation statuses in table 4, which is difficult to understand. According the table 4, why is the total number of patients without fecal incontinence (46 cases) not equal to the total number of patients with intentional defecation (11 cases)?

**Response:** In this study, the defecation of watery stool which did not pass through the TDT occurred in 16 of 51 patients in spite of TDT placement after surgery. Of the 16 patients, 11 had intentional defecation, and 5 had fecal incontinence. There was no overlap between the 11 patients and the 5 patients. We evaluated the correlation between anastomotic leakage and intentional defecation in the 11 positive and 40 negative patients and the correlation between anastomotic leakage and fecal incontinence in the 5 positive and 46 negative patients.

Q8. How many of patients had major and minor anastomotic leakage? What is the re-operation rate due to leakage?

**Response:** In the present study, of the eight instances of anastomotic leakage, major leakage occurred in two patients, and minor leakage occurred in six patients. Re-operation for anastomotic leakage was performed in the 2 major leakage patients (3.9%). Of the four anastomotic leakages during TDT placement, major leakage occurred in two patients, and minor leakage occurred in two patients. All instances of anastomotic leakage after TDT removal were minor leakages. We have now mentioned this in the Results section of the revised manuscript (P13 lines 5-10).

Q9. All patients had the standard mechanical bowel preparation on the day before surgery. So, when did the first defecation postoperatively occur?

**Response:** We found it difficult to define the first defecation, as all of the patients had TDTs placed for at least the first 5 postoperative, and watery stool was drained through the TDT. The first defecation, which we defined as ‘intentional defecation’ during TDT placement, occurred in two patients on POD 1, four patients on POD 2, one patient on POD 3, two patients on POD 4 and one patient on POD 5.

Q10. Leakage was reported in the other 4 of 51 patients after TDT was removed. However, there are again 47 patients in table 6. Where are the remaining 4 patients?

**Response:** Table 6 (Table 7 in the revised manuscript) shows the results of the subgroup analysis for 47 patients, excluding the 4 with anastomotic leakage during TDT placement. Please confirm the Methods section of the manuscript (P9 lines6-9).

Q11. The discussion section has generally superficial content. The results of the study were not sufficiently interpreted and discussed. Especially, the association between tumor diameter and fecal volume and its mechanism lack the support of the literature. Therefore, the discussion section should be revised.

**Response:** We have now added a comment concerning the association between the tumor diameter and fecal volume in the Discussion (p25 lines9-14) as follows:

“[…] a large tumor can cause stenosis of the bowel, resulting in bowel preparation not providing sufficient elimination of the intestinal contents and contents therefore being left in the bowel after laxative administration. In addition, a large postoperative fecal volume was considered to occur due to a large amount of watery stool being moved into the rectum upon the release of the stenosis by surgery and the restart of intestinal peristalsis.”

Again, we appreciate all the insightful comments. Thank you for taking the time and energy to help us improve the paper.
